# Single-Electrode Tandem Electrocatalysis with Dual TiO_2_@Cu and Cu Surfaces Enables Energy-Efficient Ammonia Production from Nitrate

**DOI:** 10.1021/acssuschemeng.5c14004

**Published:** 2026-04-23

**Authors:** Marcelo E. Chávez, Inna Y. Khairani, Martí Biset-Peiró, Sara Martí-Sánchez, Katherine Villa, Jordi Arbiol, Joan R. Morante, Bilal Gökce, Sebastián Murcia-López

**Affiliations:** † 235241 Catalonia Institute for Energy Research (IREC), Sant Adrià de Besòs 08930, Spain; ‡ University of Barcelona (UB), Barcelona 08028, Spain; § Chair of Materials Science and Additive Manufacturing, School of Mechanical Engineering and Safety Engineering, 26603 University of Wuppertal, Wuppertal 42119, Germany; ∥ Catalan Institute of Nanoscience and Nanotechnology (ICN2), CSIC and BIST, Campus UAB Bellaterra, Barcelona, Catalonia 08193, Spain; ⊥ Institute of Chemical Research of Catalonia (ICIQ-CERCA), the Barcelona Institute of Science and Technology (BIST), Av. Països Catalans, 16, Tarragona E-43007, Spain; & ICREA, Pg. Lluís Companys 23, 08010 Barcelona, Catalonia, Spain

**Keywords:** electrochemical nitrate reduction, ammonia, tandem, TiO_2_, cu, energy efficiency

## Abstract

Electrochemical ammonia synthesis from nitrate can complement a hydrogen-based energy system with a liquid, shippable energy carrier. Ammonia (NH_3_) is attractive because it serves both as an energy vector and as an essential feedstock. However, nitrate reduction is an eight-electron, proton-coupled pathway that forms multiple intermediates; the NO_3_^–^ → NO_2_^–^ step is widely viewed as rate-determining, followed by nitrite reduction and hydrogenation steps, until the formation of NH_3_. Here, we implement a tandem strategy in a flow cell using a double-sided electrode with Cu and TiO_2_@Cu surfaces that cooperatively boost the NO_3_^–^ → NO_2_^–^ and NO_2_^–^ → NH_3_ conversions. The system achieves a Faradaic efficiency toward NH_3_ of 97%, with an NH_3_ selectivity of 80%, and a productivity of 7.7 mg h^–1^ cm^–2^ (0.45 mmol h^–1^ cm^–2^), corresponding to a full-cell energy efficiency of 29%. This integrated electrode–reactor design expands the accessible performance space for the NO_3_RR by combining nitrate adsorption with selective nitrite hydrogenation. These results establish a practical route for the efficient electrochemical synthesis of NH_3_ from nitrate.

## Graphical abstract

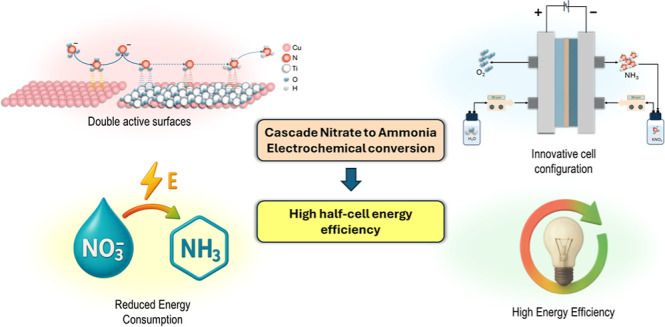

## 1 Introduction

The energy transition demands carbon-free carriers that are storable, shippable, and compatible with the existing infrastructure. Ammonia (NH_3_) is attractive because it functions both as an energy vector and as a major industrial feedstock. In addition to serving as a hydrogen carrier, NH_3_ offers a high volumetric energy density and established organization for storage and transport. Electrochemical dinitrogen reduction (e-N_2_RR) could enable sustainable NH_3_ synthesis without the energy-intensive Haber–Bosch process,^1–4^ but practical efficiencies and productivities remain low. Consequently, attention has shifted to the electroreduction of nitrogen oxyanions (NO_*x*_RR), in which nitrate (NO_3_^–^) or nitrite (NO_2_^–^) is reduced to NH_3_, offering a pathway that can simultaneously contribute to NH_3_ production and remove aqueous pollutants.

Outcomes in the NO_*x*_RR depend strongly on the electrocatalyst, operating conditions, and reactor design, which together govern intermediate binding and the local availability of NO_*x*_^–^ species. Diverse catalysts—including metals, metal oxides, single-atom catalysts, and other heterogeneous materials—have been explored to maximize NO_*x*_—to—NH_3_ conversion.^5–10^ A key challenge is suppressing competing pathways that generate NO_2_^–^, NO, N_2_O, N_2_, N_2_H_4_, and NH_2_OH.^11–14^ Most mechanistic proposals identify NO_2_^–^ and NO as semistable intermediates, motivating tandem strategies in which reduction proceeds in two sequential steps under different potentials or on complementary catalytic surfaces to steer selectivity toward NH_3_.^15,16^

Because NO_2_^–^ mediates the eight-electron pathway, maximizing NH_3_ formation requires architectures that pair efficient nitrate activation with selective nitrite conversion under minimal mass transport limitations. In the NO_3_^–^RR to NH_3_, the 2e^–^ + 6e^–^ cascade directly governs specific energy consumption and thus both half-cell and full-cell energy efficiency (EE). Only a few tandem NO_3_RR systems have been reported, typically employing complex bimetallic motifs with dual sites to promote the reaction of NO_3_^–^ → NO_2_^–^ → NH_3_ at low overpotentials. In most cases, Cu facilitates the initial NO_3_^–^ → NO_2_^–^ step, while metals such as Co or Ru,^17,18^ which exhibit stronger NO_2_RR → NH_3_ activity, drive subsequent hydrogenation. Parallel efforts have focused on developing materials to promote H* generation by accelerating water dissociation. This approach favors *NO hydrogenation, further delivering high NH_3_ productivities with heterostructures like NiNNi_3_/Cu^19^ and Ru/Co­(OH)_2_-CoP.^20^

Tandem strategies can also be realized through spatially segmented designs. Macroscopically separated regions are simpler to fabricate and can offer improved durability because the performance does not rely on preserving nanoconfined structures over long operations. Such segmentation has proven effective in tandem CO_2_ reduction,^21^ yet for NO_3_^–^RR—especially in flow-cell reactors, configurations using separated electrodes or deliberately segmented surfaces remain largely unexplored.

Here, we propose a tandem NO_3_^–^RR approach that integrates dual-surface electrodes into a modified flow cell. We select Cu and TiO_2_ as complementary active materials: beyond Co and Ru, titanium-based materials show high NH_3_ selectivity owing to strong adsorption of the *NO_2_^–^ intermediate,^22,23^ and in TiO_2_, nonintrinsic defects such as oxygen vacancies further enhance activity toward both NO_3_^–^RR and NO_2_^–^RR.^24,25^ As illustrated in Figure 1, the first surface is bare Cu, and the second comprises TiO_2_ nanoparticles supported on Cu, enabling a cascade reduction in which nitrate activation and nitrite hydrogenation occur on distinct faces. We evaluate Faradaic efficiency, product selectivity among N-containing species, and preliminary scale-up metrics for alkaline NO_3_^–^RR. By combining reactor engineering with catalysts of dissimilar intrinsic properties and optimized operating conditions, we achieve—to the best of our knowledge—the highest reported cell EE with reduced specific energy consumption for NH_3_ generation via NO_3_^–^ reduction.^26^

**Figure 1:**
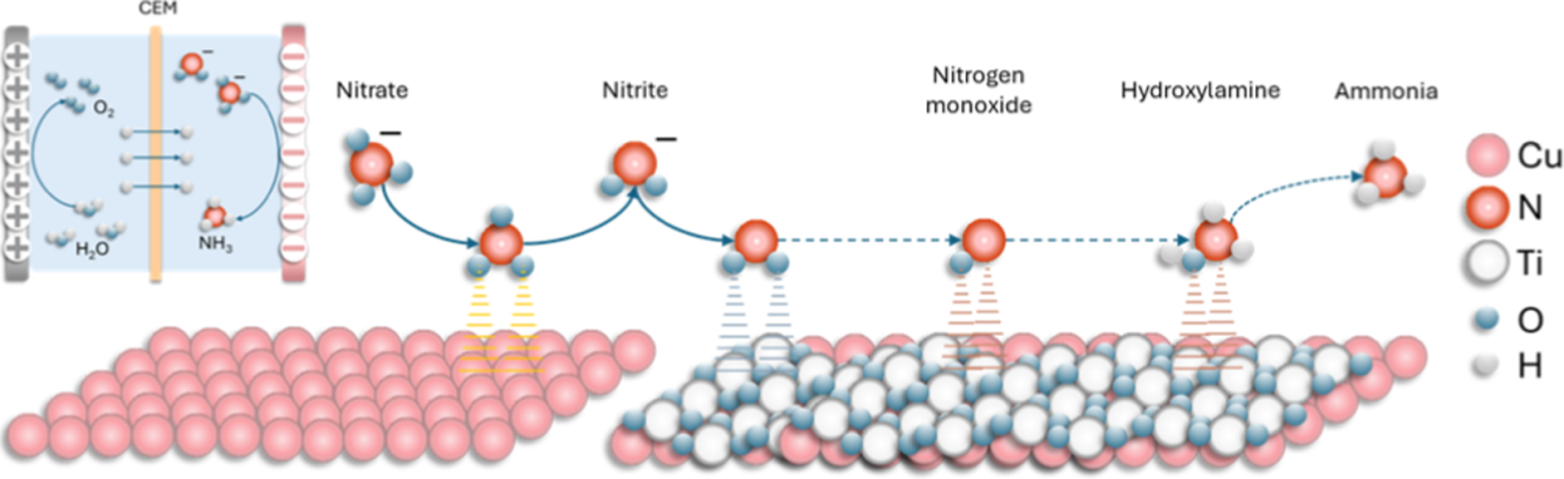
Cascade NO_3_^–^ to NH_3_ process involving a 2 + 6 electron transfer. First stage (2-electron transfer): NO_3_^–^ to NO_2_^–^ using a Cu-based active support. Second stage (6-electron transfer): NO_2_^–^ to NH_3_ was carried out using a TiO_2_@Cu catalyst.

## 2 Experimental Section

### 2.1 Chemical and Materials

Copper foil (Cu) and sodium carbonate (Na_2_SO_4_) were obtained from Thermo Scientific Chemicals (Alfa-Aesar). Potassium hydroxide (KOH) and ethanol (C_2_H_6_O) were purchased from Sigma-Aldrich. Potassium nitrate (KNO_3_), potassium nitrite (KNO_2_), Nessler’s reagent, ammonium chloride (NH_4_Cl), and sodium hydrogen carbonate (NaHCO_3_) were supplied by Merck. A titanium foil target (1 mm thick foil, 99.6% purity) was purchased from MaTecK Material-Technologie & Kristalle GmbH.

### 2.2 Synthesis of TiO_2_ Nanoparticles

Titania nanoparticles (TiO_2_ NPs) were fabricated by pulsed laser ablation in liquid (PLAL method), as it offers catalytic enhancement due to the formation of defect-rich NPs.^27,28^ A titanium target was ablated using a 1064 nm neodymium-doped yttrium aluminum garnet (Nd:YAG) laser with a pulse duration of 10 ps, a repetition rate of 1000 kHz, and a laser power of 43 W. The laser was coupled to a galvanometer scanner (scanning speed 20 m/s) and an f-theta lens (focal length 167 mm) to steer and focus the laser beam into an Archimedean spiral pattern. The PLAL process was performed in a flow chamber^29–31^ with ethanol as the liquid carrier, selected for its role as a capping agent to hinder the nanoparticle growth (size quenching).^32^ The resulting colloidal dispersion of TiO_2_ NPs in ethanol was partially evaporated until it achieved a concentration of 0.125 mg mL^–1^.

### 2.3 TiO_2_@Cu Electrode Preparation

Copper (Cu) supports were pretreated to remove surface oxides. Cu foil was sonicated for 15 min in a 1:1:1 mixture of acetone, isopropanol, and ethanol, followed by immersion in an acidic solution (10% H_2_SO_4_ + 36 g L^–1^ C_6_H_8_O_7_) under sonication for 5 min. After thorough rinsing with Milli-Q water for 10 min, the catalyst ink was prepared using the TiO_2_ dispersion described in Section 2.2. The dispersion was diluted to 0.0625 mg mL^–1^ with anhydrous ethanol, and 2 wt % Nafion perfluorinated solution was added as a binder. The final ink consisted of 0.50 mL of TiO_2_ dispersion (0.125 mg mL^–1^), 0.48 mL of ethanol, and 0.02 mL of binder solution. This ink was drop-cast onto a pretreated Cu substrate (1 cm^2^ area) and dried in a vacuum oven for 3 h. The resulting electrodes contained 0.0625 mg of cm^–2^ TiO_2_. The fabrication process is illustrated in Figure S1.

### 2.4 Electrode Characterization

The morphology of TiO_2_ nanoparticles and the Cu surface was examined by using a field emission scanning electron microscope (Zeiss Auriga series). Elemental composition was determined by energy-dispersive X-ray spectroscopy (EDX). X-ray diffraction (XRD) patterns were collected on a Bruker D8 Advanced diffractometer using Cu Kα radiation (λ = 1.5418 Å) to analyze the crystallographic structures. The crystal structure of the nanoparticles was analyzed by means of high-resolution transmission electron microscopy (HRTEM) by using a FEI Tecnai F20 TEM with a field emission gun. The TiO_2_ structure was also analyzed by Raman spectroscopy with an iHR320 spectrometer from HORIBA Scientific with a green laser (λ = 532 nm). X-ray photoemission spectroscopy (XPS) was employed to prove the chemical and electronic states of the electrode components, using a SPECS system equipped with an XR50 source operating at 300 W and a Phoibos 150 MCD-9 detector with an Al Kα source. The pass energy of the hemispherical analyzer was set at 20 eV, and the energy step of the high-resolution spectra was 0.1 eV.

Continuous wave electron paramagnetic resonance (EPR) spectra of TiO_2_ were acquired on a Bruker EMX Micro X-band spectrometer operating at 9.85 GHz using a Bruker ER 1164 HS resonator. Individual capillaries were filled with the samples and were placed into EPR tubes at the same position of the resonant cavity for EPR spectral acquisition. The spectral data were collected at room temperature with the following spectrometer settings: microwave power = 0.6479 mW; center field = 3200 G, sweep width = 6000 G, sweep time = 60 s, modulation frequency = 100 kHz, modulation amplitude = 4 G, power attenuation = 25 dB, and time constant = 0.01 ms.

### 2.5 Electrochemical Measurements

All electrochemical experiments were performed at room temperature using a BioLogic electrochemical workstation. Three-electrode cell tests with pristine and TiO_2_-loaded Cu disk electrodes were conducted for NO_3_^–^RR and NO_2_^–^RR studies in 0.1 M KNO_3_ + 1 M KOH, 0.1 M KNO_2_ + 1 M KOH, and 1 M KOH electrolytes. A Hg/HgO electrode served as the reference electrode and a Pt mesh as the counter electrode (CE). Prior to each experiment, the electrolyte was purged with Ar for 20 min. Linear sweep voltammetry (LSV) was conducted at a scan rate of 20 mV s^–1^.

A flow-cell configuration was employed for the chronoamperometry and chronopotentiometry tests. Nafion-117 membrane separates the cathode and anode compartments. To capture NH_3_ evolved during electrolysis, a gas absorption chamber filled with 0.01 M H_2_SO_4_ was connected to both catholyte and anolyte bottles. Ar was circulated through the headspace of the two bottles. Total NH_3_ content (catholyte, anolyte, and trap) was used for the yield calculations. The flow-cell configurations are shown in Figure S2.

Pristine Cu and TiO_2_@Cu electrodes were used as working electrodes (WEs), while leak-free Ag/AgCl and a commercial dimensionally stable anode (DSA) were employed as a reference electrode (RE) and CE, respectively.

Chronoamperometry and chronopotentiometry tests were performed for the NO_3_^–^RR and the NO_2_^–^RR under various conditions (*E*_W_ = −0.4 to −0.8 V vs RHE; *j* = 35 to 110 mA cm^–2^). Faradaic efficiencies (FE_i_) toward NH_3_, NO_2_^–^, and H_2_ and selectivities (SE_i_) toward NH_3_ and NO_2_^–^, and NH_3_ yield (productivity) were evaluated as direct efficiency parameters. Full cell EE (EE_cell_), half-cell EE (EE_half‑cell_), and energy consumption per mass of NH_3_ or NO_3_^–^ ( $EC_{NH_{3}}andEC_{NO_{3}^{-}}$ respectively) were also evaluated. Equations 1–7 define all metrics^33–35^

1 $$FE_{i}\left(\%\right)=\frac{\alpha \cdot C_{i}\cdot F}{M\cdot Q}\cdot 100$$

2 $$NH_{3}yield=\frac{C_{NH_{3}}\cdot V\cdot M}{A\cdot t}$$

3 $$SE_{i}\left(\%\right)=\frac{C_{i}}{C_{NO_{3_{0}}^{-}}\cdot C_{NO_{3_{t}}^{-}}}\cdot 100$$

4 $$EE_{cell}\left(\%\right)=\frac{\left|EC_{NH_{3}}\right|}{\left|E_{CELL}\cdot Q\right|}\cdot 100$$

5 $$EE_{half‐cell}\left(\%\right)=\frac{EC_{NH_{3}}}{\left(0.4-E_{w}\right)\cdot Q}\cdot 100$$

6 $$EC_{NH_{3}}=\left[\Delta G_{NH_{3}}^{0}+R\cdot T\cdot \ln\left(\frac{{C_{NH_{3}}}_{t}\cdot {C_{OH^{-}}}_{t}^{9}}{{C_{NO_{x}^{-}}}_{t}}\right)\right]\cdot {C_{NH_{3}}}_{t}\cdot V$$

7 $$EC_{NO_{3}^{-}}=\frac{\left|E_{CELL}Q\right|}{m_{NO_{3}^{-}}}$$

where *C*_i_ (M) is the molar concentration of a target product or evaluated reactant; *V* (L) is the volume of the electrolyte; *M* (g·mol^–1^) is the molar mass of the target product or reactant; *A* (cm^2^) is the geometric surface area of the electrode; α is the number of transferred electrons to produce a defined product; *F* is the Faradaic constant expressed in SI units; *Q* is the total transferred charge to the system; *C*_NH_3__(M) is the measured ammonia concentration at the end of NO_3_^–^RR and NO_2_^–^RR; *t* (h) is the electrolysis time; $C_{NO_{3_{0}}^{-}}$ and $C_{NO_{3_{t}}^{-}}$ (M) represent the initial and final concentration of NO_3_^–^ ion in the electrolyte. $\Delta G_{NH3}^{0}$ (J or kWh) is the standard Gibbs free energy to form NH_3_ from NO_3_^–^ or NO_2_^–^; *E*_cell_ (V) is the applied cell potential to carry out the reaction; *E*_W_ (V) represents the applied potential; *m*_NH_3__ (kg) is the mass of NH_3_ produced during electrolysis, and $m_{NO_{3}^{-}}$ (kg) is the mass of NO_3_^–^ consumed during the electrolysis.

### 2.6 Analytical Instrumentation and Measurements

Hydrogen (H_2_) was analyzed by using a gas chromatography system (GC, Agilent Technologies 490 Micro-GC). Nitrate and nitrite concentrations were determined by ion chromatography (DIONEX 1100) with a Dionex Ion Pack AS-22 anion exchange column and a chemical suppressor (ASR-ultra 4 mm). The eluent (4.5 mM Na_2_CO_3_ + 1.4 mM NaHCO_3_) was delivered at a flow rate of 1.5 mL min^–1^. Ammonia (NH_3_/NH_4_^+^) was quantified by UV–vis spectrophotometry (PerkinElmer Lambda 950) after complexation of NH_4_^+^ ions with Nessler reagent.^36^ Calibration curves are shown in Figure S3.

## 3 Results and Discussion

### 3.1 TiO_2_@Cu Characterization

Figure 2a shows the XRD patterns of the Cu substrate and the TiO_2_@Cu electrodes. For the Cu substrate, four characteristic peaks of face-centered cubic Cu (JCPDS 04-0836) are observed: two intense peaks at 2θ = 43.3° and 73.99°, corresponding to the (111) and (220) planes, and two less pronounced peaks at 50.4° and 90.2°, assigned to the (200) and (300) planes.^37^ The TiO_2_@Cu electrode exhibits the same Cu-related peaks, indicating minimal changes in the Cu-based component. No diffraction peaks associated with Ti-containing phases are detected, which is consistent with the small size of the TiO_2_ nanoparticles. Additional structural characterization by Raman spectroscopy is shown in Figure 2b. Five distinct Raman active modes characteristic of anatase TiO_2_ were identified at frequencies of 143, 193, 392, 510, and 639 cm^–1^, corresponding to the symmetries of Eg, Eg, B1g, A1g, and Eg, respectively. These vibrational frequencies and their relative intensities confirm the anatase phase of TiO_2_.^38^

**Figure 2:**
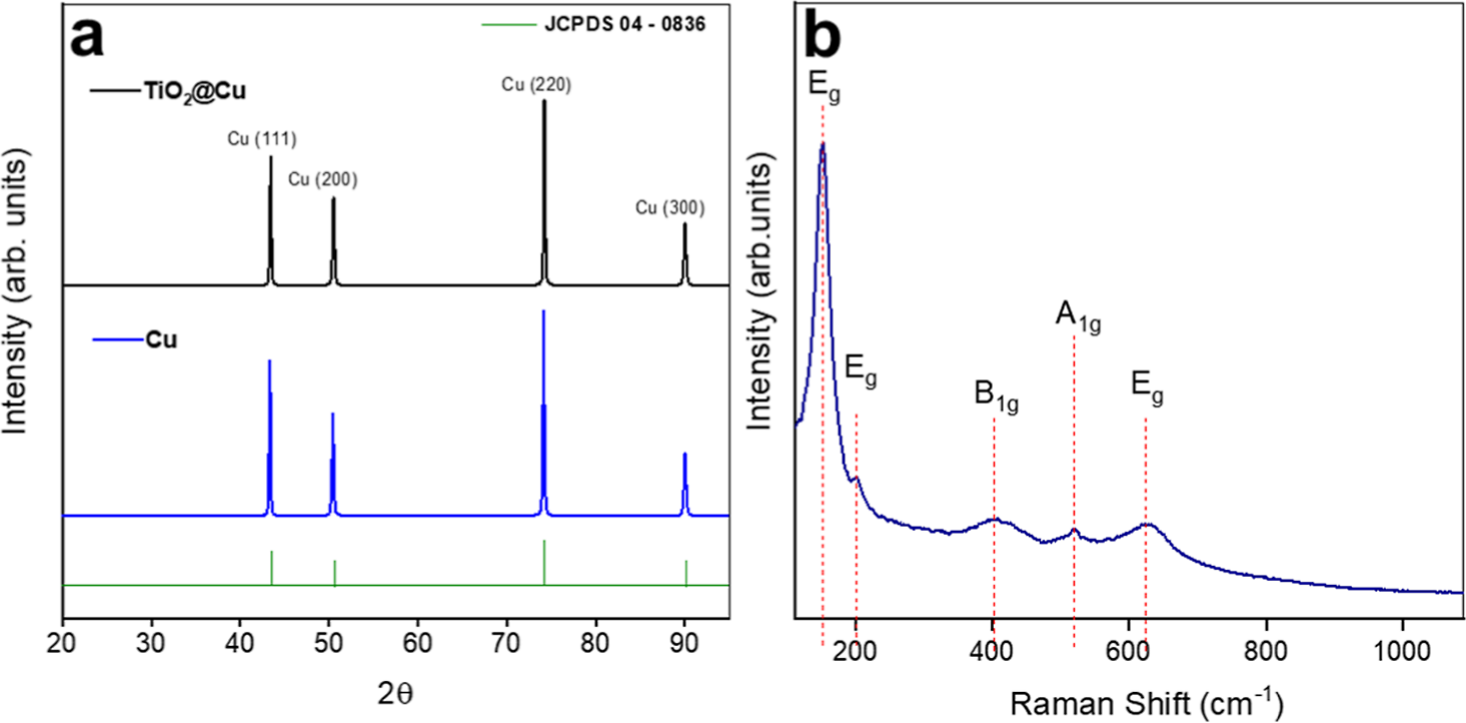
(a) XRD patterns for the Cu and TiO_2_@Cu electrodes; (b) Raman spectrum of TiO_2_ nanoparticles with the corresponding anatase Raman shifts.

HR-TEM analysis of the TiO_2_ nanoparticles is included in Figure 3a–e. The particles display a range of particle sizes, predominantly a few nanometers in diameter, and crystallize in the anatase tetragonal phase of TiO_2_, which has also been confirmed by the *d*-spacing measured in Figure 3d, corresponding to the (101) and (105)/(112) lattice planes of anatase.

**Figure 3:**
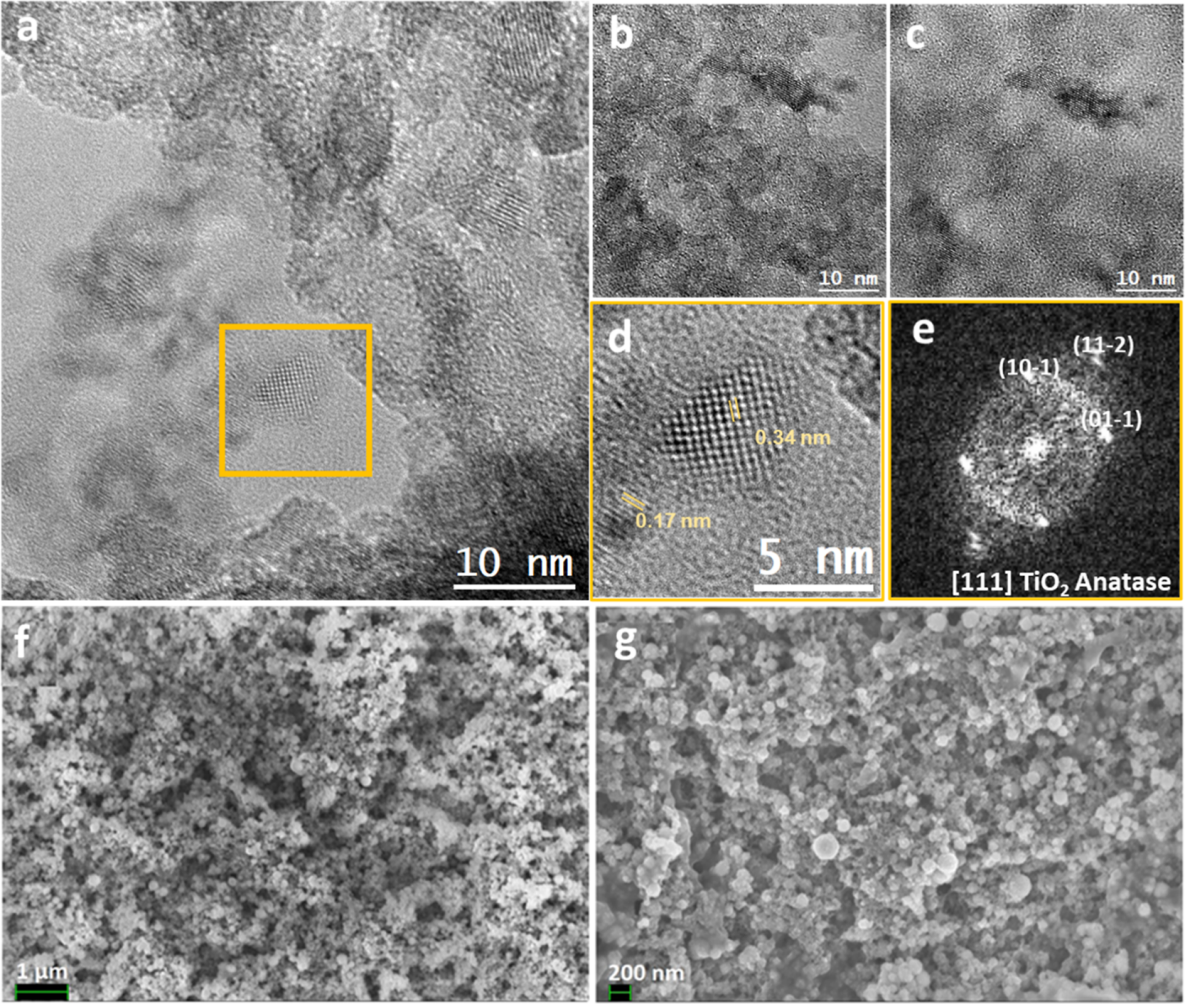
TEM analyses of TiO_2_ nanoparticles: (a,c) HRTEM general views of the nanoparticles. (d) HRTEM magnified detail of the squared region in (a); (e) corresponding indexed power spectra of the magnified detail in (d). (f,g) SEM general view images of the TiO_2_@Cu electrode.

SEM images of the TiO_2_@Cu electrode are presented in Figures 3f,g, revealing aggregated porous layers on the electrode surface both before (Figure 3f) and after (Figure 3g) electrolysis. EDX analysis shows an estimated atomic composition of 4.3% Ti, 35.2% Cu, and 20.8% O, along with notable amounts of C and F originating from the Nafion ionomer. Elemental mapping (Figure S5) confirms the uniform distribution of these elements, with O, F, and C exhibiting similar spatial distribution patterns to Ti.

The presence of oxygen vacancies in TiO_2_ was determined by XPS and EPR analyses. Figure 4 presents the XPS spectra of the main components of the TiO_2_@Cu electrodes before and after electrolysis. In the high-resolution Cu 2p spectrum (Figure 4a), peaks at 933.2 and 955.9 eV correspond to the 2p_3/2_ and 2p_1/2_ sublevels of Cu^0^/Cu^+^. Additional satellite peaks at 943.2 and 964.2 eV, typically associated with surface Cu^2+^, are also observed.^11,33,34^ After electrolysis (Figure 4b), the intensity of these satellite peaks decreases, indicating a partial electrochemical reduction of surface Cu^2+^. Both Cu_2_O and CuO have been reported to exhibit electrocatalytic activity for NO_3_^–^-to-NH_3_ conversion.^33,34^ Postelectrolysis analysis in Figure S5 shows no evidence of bulk Cu oxidation in the TiO_2_@Cu electrode according to the XRD results.

**Figure 4:**
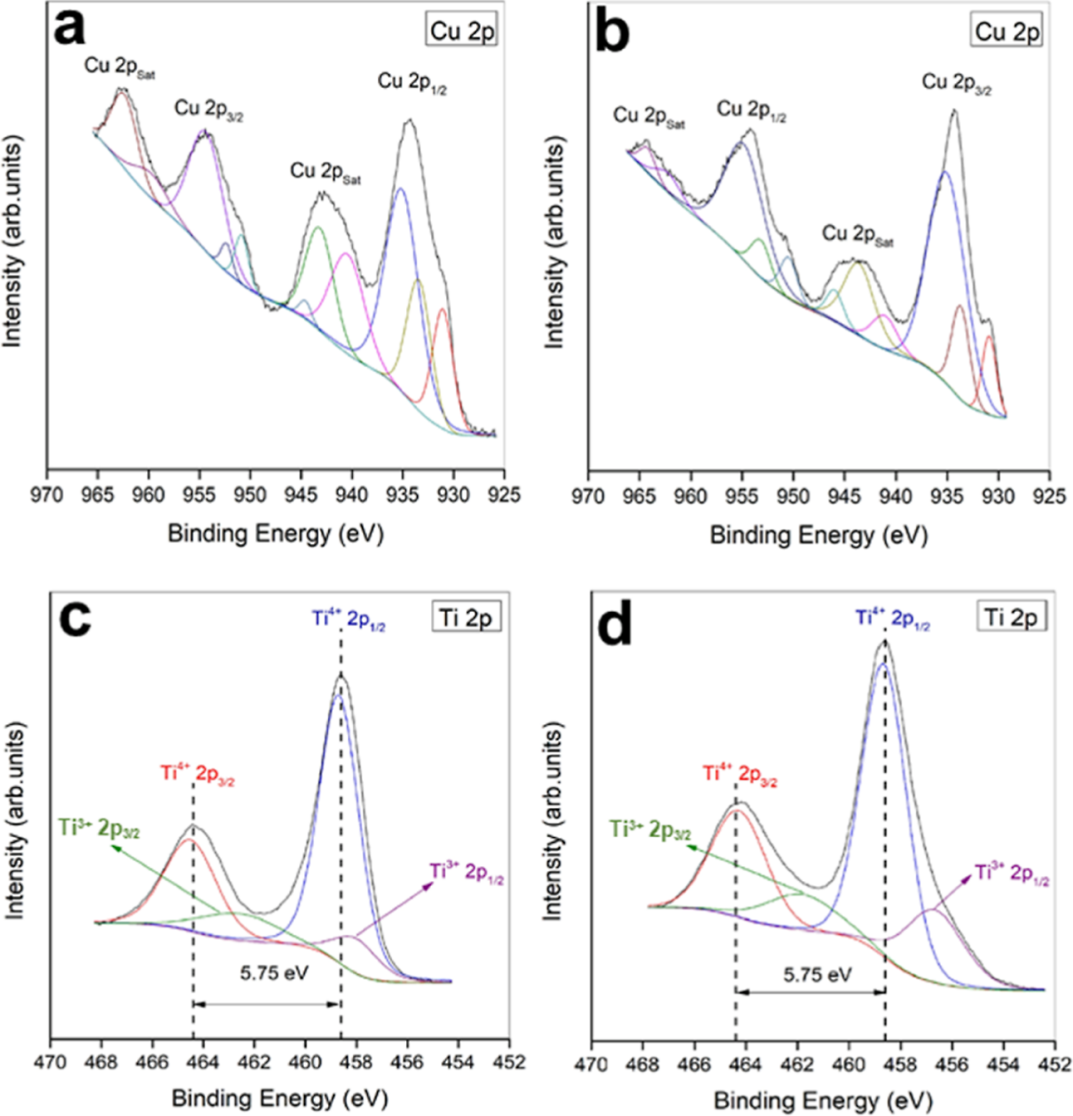
XPS analysis of the TiO_2_@Cu electrode: (a) before and (b) after electrolysis at the Cu 2p level; (c) before and (d) after electrolysis at the Ti 2p level.

Figure 4c,d displays the high-resolution Ti 2p spectra before and after electrolysis, showing four distinct peaks assigned to titanium dioxide (Ti^4+^) and titanium subdioxide (Ti^3+^) in the 2p_3/2_ and 2p_1/2_ sublevels, respectively.^39^ Specifically, Ti^4+^ 2p_1/2_ appears at 464.4 eV, Ti^4+^ 2p_3/2_ at 458 eV, Ti^3+^ 2p_1/2_ at 461.8 eV, and Ti^3+^ 2p_3/2_ at 460.2 eV. The separation of 5.75 eV between each pair of 2p_3/2_ and 2p_1/2_ peaks matches the standard binding energy difference for TiO_2_. The detection of Ti^3+^ peaks confirms the presence of oxygen vacancies on the surface,^40^ further supported by the EPR spectrum shown in Figure S6. A distinct resonance signal is observed with a *g*-value of 2.0018, which can be attributed to Ti^3+^ centers formed as a result of oxygen deficiency. These Ti^3+^ species arise when oxygen vacancies trap electrons, leading to the reduction of neighboring Ti^4+^ to Ti^3+^. The obtained g-value is in agreement with the reported values for oxygen-deficient TiO_2_, confirming that the nanoparticles contain oxygen-related defects.^41,42^

The preliminary electrochemical characterization of TiO_2_@Cu for the NO_3_^–^RR and NO_2_^–^RR was conducted in a three-electrode cell. TiO_2_ nanoparticles were deposited onto a polished copper disk, and LSV was performed in three electrolytes: 0.1 M KNO_3_ + 1 M KOH for the NO_3_^–^RR, 0.1 M KNO_2_ + 1 M KOH for the NO_2_^–^RR, and 1 M KOH as a blank. Four distinct peaks (P_1_–P_4_) are identified in Figure 5: P_1_ between 0 and 0.1 V vs RHE, P_2_ between −0.2 and −0.1 V vs RHE, P_3_ between −0.4 and −0.35 V vs RHE, and P_4_ between −0.55 and −0.5 V vs RHE. Peak P_1_ exclusively appeared during NO_3_^–^RR and corresponds to the RDS, a two-electron transfer reaction associated with the conversion of NO_3_^–^ to NO_2_^–^.^43^ Peak P_2_, observed for both NO_3_^–^RR and NO_2_^–^RR, is assigned to a four-electron transfer reducing NO_2_^–^ to hydroxylamine (NH_2_OH),^44^ which can subsequently be reduced to NH_3_. Peak P_3_, also present in both reactions, corresponds to the six-electron reduction of NO_2_^–^ to NH_3_, in agreement with previous reports.^44,45^ No discernible peaks were detected in the blank electrolyte, confirming that these electrochemical features arise specifically from the NO_3_^–^ and NO_2_^–^ species.

**Figure 5:**
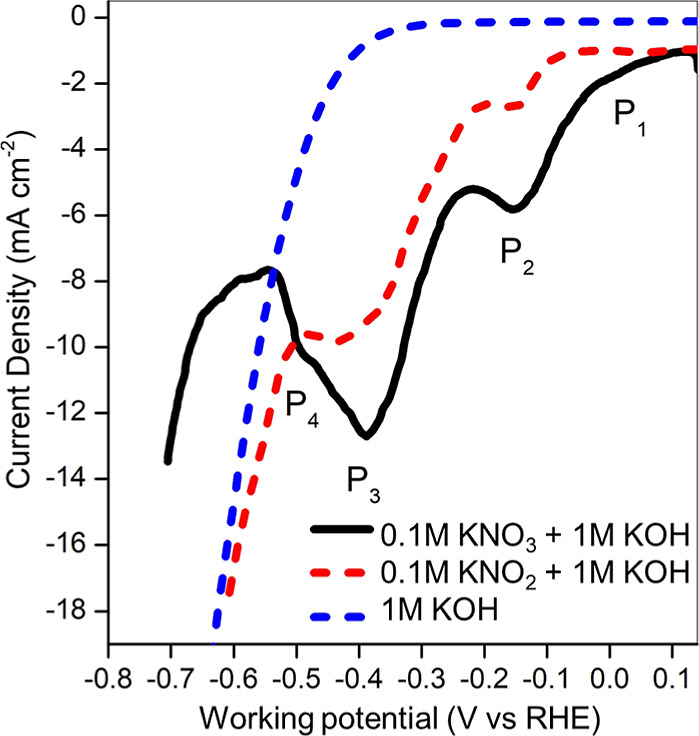
Preliminary electrochemical characterization of TiO_2_ deposited on a Cu disk electrode in a three-electrode cell. Linear scan voltammetries under different electrolyte compositions.

During NO_3_^–^RR, the current densities for P_2_ and P_3_ were higher than in NO_2_^–^RR. This can be attributed to the elevated interfacial concentration of NO_2_^–^ generated in situ during the RDS, which shows limited desorption from the Cu-based electrodes,^46^ thereby reducing diffusion limitations. In contrast, during the NO_2_^–^RR, NO_2_^–^ ions must diffuse from the bulk electrolyte to the electrode surface.

The increased HER overpotential observed for NO_3_^–^RR aligns with mechanistic insights from previous studies.^16,47^ These studies show that the NO_3_^–^RR involves sequential deoxygenation and hydrogenation steps mediated by adsorbed hydrogen (*H). The availability of *H depends on NO_3_^–^ concentration, since all nitrogen intermediates formed from NO_3_^–^ react with *H until NH_3_ is produced. At lower overpotentials (P1), the sluggish generation rate of *H favors the accumulation of non-hydrogenated intermediates such as NO_2_^–^, regardless of $C_{NO_{3}^{-}}$ . At higher overpotentials (P_3_ or P_4_), the rapid generation of *H promotes further hydrogenation of nitrogen intermediates, particularly at high $C_{NO_{3}^{-}}$ . As a result, the *H balance is maintained, and competitive HER is suppressed.

### 3.2 Catalytic Performance of Individual Electrodes

The flow cell (Figure S2a) was assembled using 1 cm^2^ TiO_2_@Cu or pristine Cu as WE, a DSA serving as CE, and an Ag/AgCl RE. To compare the individual performance of bare Cu and TiO_2_@Cu for NO_3_^–^RR and NO_2_^–^RR, chronoamperometry tests were performed for 2 h at each potential in 0.1 M KNO_3_ + 1 M KOH (NO_3_^–^RR) and 0.1 M KNO_2_ + 1 M KOH (NO_2_^–^RR). For NO_3_^–^RR, only FE toward NO_2_^–^ and the specific nitrate conversion per unit charge ( $\Delta C_{NO_{3}^{-}}$ ) were determined.

The NO_3_^–^RR results (Figure 6a) show that both Cu and TiO_2_@Cu electrodes display decreasing ${FE}_{NO_{2}^{-}}$ values at more negative potentials, with Cu consistently achieving higher values. The maximum FE of 65% was obtained at −0.4 V vs RHE for Cu, decreasing to below 10% at potentials more negative than −0.7 V vs RHE. Previous studies have linked high overpotentials to enhanced HER, particularly on materials with weak hydrogen adsorption, which limits NO_3_^–^RR. At lower overpotentials, cleavage of N–O bonds (deoxygenation) in adsorbed NO_2_^–^ becomes rate-limiting due to insufficient H_ads_, leading to NO_2_^–^ release and accumulation in the bulk electrolyte.^48,49^ In terms of NO_3_^–^ conversion, Cu again outperformed TiO_2_@Cu, reaching $\Delta C_{NO_{3}^{-}}$ = 0.6 $mg_{NO_{3}^{-}}$ C^1–^ at −0.4 V vs RHE. TiO_2_@Cu followed similar trends of decreasing specific conversion and ${FE}_{NO_{2}^{-}}$ at higher overpotentials but with lower values across all conditions.

**Figure 6:**
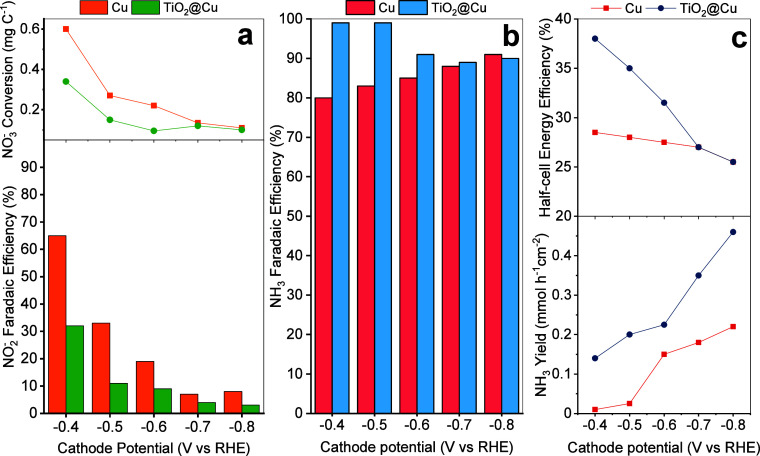
Electrocatalytic activity of the single-side TiO_2_@Cu and Cu electrodes: (a) specific NO_3_^–^ conversion and Faradaic efficiency to NO_2_^–^ during NO_3_^–^RR-to-NO_2_^–^ tests; (b) Faradaic efficiency to NH_3_; and (c) EE_half‑cell_ and NH_3_ productivity in NO_2_^–^RR-to-NH_3_ tests.

For NO_2_^–^RR to NH_3_ (Figure 6b,c), TiO_2_@Cu achieved FE_NH_3__ of 99% at −0.4 V and −0.5 V vs RHE. As the potential became more cathodic, the values gradually decreased and stabilized around 90%. In contrast, Cu exhibited the opposite trend, with FE_NH_3__ increasing from 80% at −0.4 V to 90% at −0.8 V vs RHE. The incorporation of TiO_2_ into Cu thus lowers the overpotential, enabling high FE_NH_3__ under relatively mild cathodic conditions. In alkaline media, *H species originate from water dissociation, making this process a critical step in subsequent hydrogenation reactions.^20^ Computational studies have extensively investigated Cu, TiO_2_, and bimetallic surfaces in this context. On Cu surfaces, water dissociation generally exhibits moderate barriers, strongly influenced by the degree of water coverage. Accordingly, surface properties such as oxophilicity have been identified as key parameters for enhancing water binding and promoting water dissociation.^19,50,51^ In contrast, studies on rutile- and anatase–water interfaces indicate a certain probability of spontaneous dissociative water adsorption, particularly on defective surfaces.^52,53^ Consequently, the lower FE observed for Cu at less negative potentials can be attributed to the decreased hydrogen adsorption, resulting in low hydrogen coverage and a slower *NH_*x*_ hydrogenation rate.^54^

Across the entire potential range, TiO_2_@Cu also delivered higher EE_half‑cell_ values than Cu, starting at 38% at −0.4 V vs RHE and gradually decreasing to 28% at −0.8 V vs RHE (calculation details are given in Supporting Information). NH_3_ productivity for the NO_2_^–^RR (Figure 6c) increased with potential, reaching 0.45 mmol h^–1^ cm^–2^ for TiO_2_@Cu and 0.21 mmol h^–1^ cm^–2^ for Cu under optimal conditions. These results demonstrate the superior NO_2_^–^RR electrocatalytic performance of TiO_2_@Cu over bare Cu at low overpotentials.

### 3.3 Cell Configuration: Tandem Approach

Building on the previous results where Cu outperformed TiO_2_@Cu for the initial NO_3_^–^RR to NO_2_^–^, while TiO_2_@Cu was more efficient for the NO_2_^–^RR to NH_3_ (Figure 6a), we propose a tandem electrocatalytic system designed to cascade the initial accumulation of NO_2_^–^ intermediates into their final conversion to NH_3_ at lower overpotentials. For this purpose, the electrochemical cell was adapted to expose two faces (each with a 1 cm^2^ geometric area). This configuration leverages the individual strengths of each catalyst while mitigating their respective limitations. Surface 1 consisted of bare Cu, selected for its superior activity in the RDS of NO_3_^–^ reduction.^55^ In this role, Cu facilitates the generation and desorption of NO_2_^–^ as the first intermediate in the cascade pathway.^42^ This partial reduction of NO_3_^–^ to NO_2_^–^ sets the stage for the subsequent conversion to NH_3_. Surface 2 consisted of TiO_2_@Cu, which was responsible for driving the NO_2_^–^RR (and NO_3_^–^RR) toward NH_3_ formation. This second stage is optimized for lower overpotentials, with the added benefit of suppressing the HER.

The cathode arrangement and cell layout are illustrated in Figure 7. By pairing Cu and TiO_2_@Cu in this tandem configuration, the system aims to maximize the NO_3_^–^ and NO_2_^–^ conversions at lower overpotentials, thereby improving the overall EE. The performance of this setup was evaluated by chronoamperometry tests across a range of applied potentials in a flow-cell configuration. In all tests, both electrolytes consisted of 0.1 M KNO_3_ + 1 M KOH aqueous solutions.

**Figure 7:**
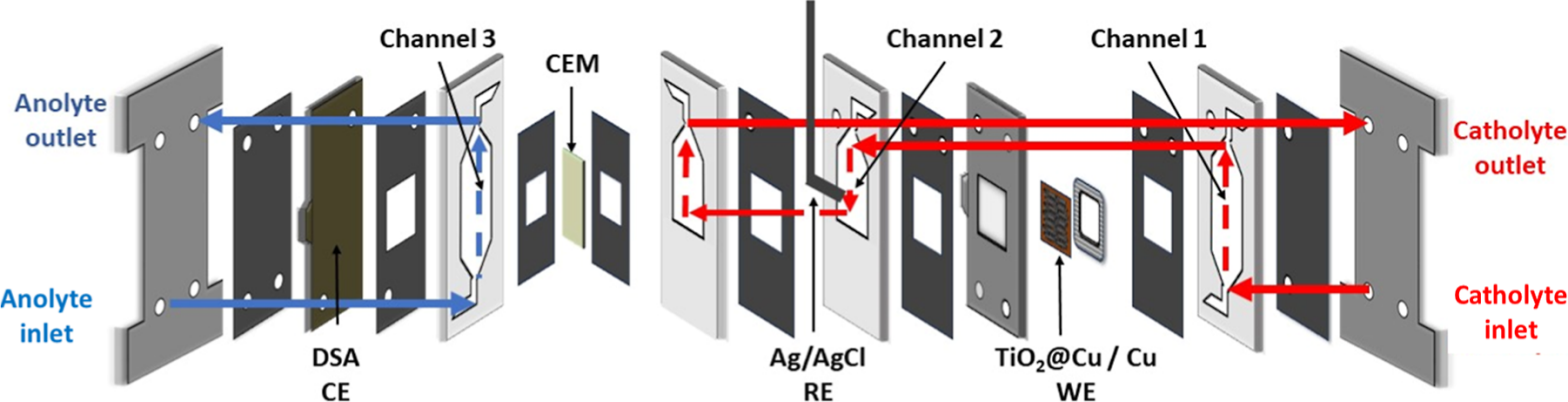
Three-channel electrochemical flow cell adapted with a current collector that exposes two sides of the WE.

Figure 8a shows the FE distribution for the tandem configuration over the applied potential range. Across all potentials, NH_3_ was the predominant product. A volcano-shaped trend was observed, with the maximum FE_NH_3__ (91%) at −0.6 V vs RHE, and values remaining above 85% at −0.4 V, −0.5 V, and −0.7 V vs RHE. In contrast, ${FE}_{NO_{2}^{-}}$ decreased progressively with increasing overpotential, dropping from 13% at −0.4 V to 3% at −0.8 V vs RHE. The remaining FE toward other products aligned with HER trends as determined by GC analysis. Importantly, FE toward H_2_ was significantly suppressed by the high bulk concentrations of NO_3_^–^ and K^+^ ions, as well as by the presence of NO_2_^–^ intermediates, whose faster reduction kinetics outcompete H_2_O reduction to H_2_.^56,57^ Previous studies have also reported more favorable *NO_2_ hydrogenation in the presence of O.V., which can effectively inhibit the formation of undesired nitrogenated byproducts.^42^ These findings are consistent with the selectivity trends in Figure 8b: peak selectivity toward NH_3_ was achieved at −0.4 V vs RHE (86%), followed by a gradual decline at more cathodic potentials, although it remained above 80% across the studied potential range. Conversely, the selectivity toward NO_2_^–^ decreased steadily, reaching its maximum of 14% at −0.5 V vs RHE.

**Figure 8:**
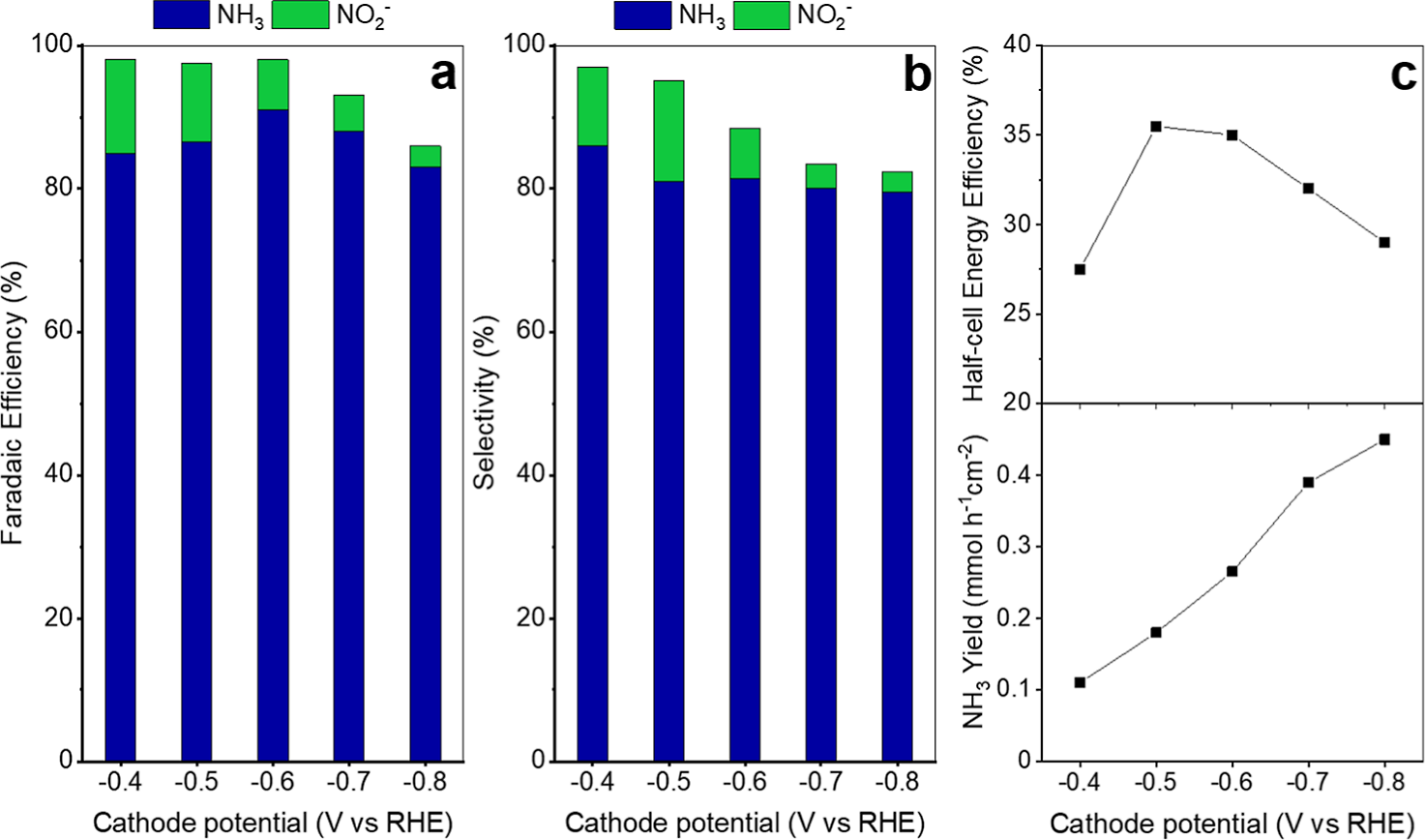
NO_3_^–^RR to NH_3_ for the tandem configuration: (a) FE distribution of products, (b) SE of products, and (c) EE_half‑cell_ and NH_3_ productivity as a function of *E*_W_.

Figure 8c presents the corresponding EE_half‑cell_ values. These closely follow the FE_NH_3__ tendency, exceeding 35% at −0.5 V and −0.6 V vs RHE, being among the highest half-cell energy efficiencies reported.^17,44^ At higher overpotentials, EE_half‑cell_ declined, likely due to the increasing contribution of the HER to the overall energy consumption. The NH_3_ yield increased with applied potential, approaching a plateau at −0.8 V vs RHE, where it reached 0.45 mmol h^–1^ cm^–2^. Although higher yields were attainable at more negative potentials, the volcano-shaped FE_NH_3__ indicates a reduced conversion efficiency of NO_3_^–^ to NH_3_ under these conditions.

### 3.4 Energy Analysis—Increased Cell EE

To further assess the advantages of the tandem configuration, comparative tests were performed using single- and double-sided TiO_2_@Cu cathodes (1 and 2 cm^2^ geometric areas, respectively). Figure 9a presents the estimated EE_cell_ values, which exhibited a characteristic volcano-shaped trend for all three configurations. The tandem system delivered the highest efficiencies, reaching 28.7 and 28.5% at −0.5 V and −0.6 V vs RHE, respectively, highlighting the efficient utilization of supplied energy for conversion of NO_3_^–^ to NH_3_. At higher cathodic potentials, EE_cell_ gradually decreased, reaching a minimum of 23% at −0.8 V vs RHE, due to the growing influence of HER and other side reactions. Both 1 cm^2^ and 2 cm^2^ TiO_2_@Cu electrodes exhibited lower EE_cell_ values, with maxima centered at −0.5 V vs RHE. The slightly higher EE_cell_ observed for the 1 cm^2^ TiO_2_@Cu compared to the 2 cm^2^ version can be attributed to the cell disposition (Figure 7), in which the reference electrode faces only surface 2.

**Figure 9:**
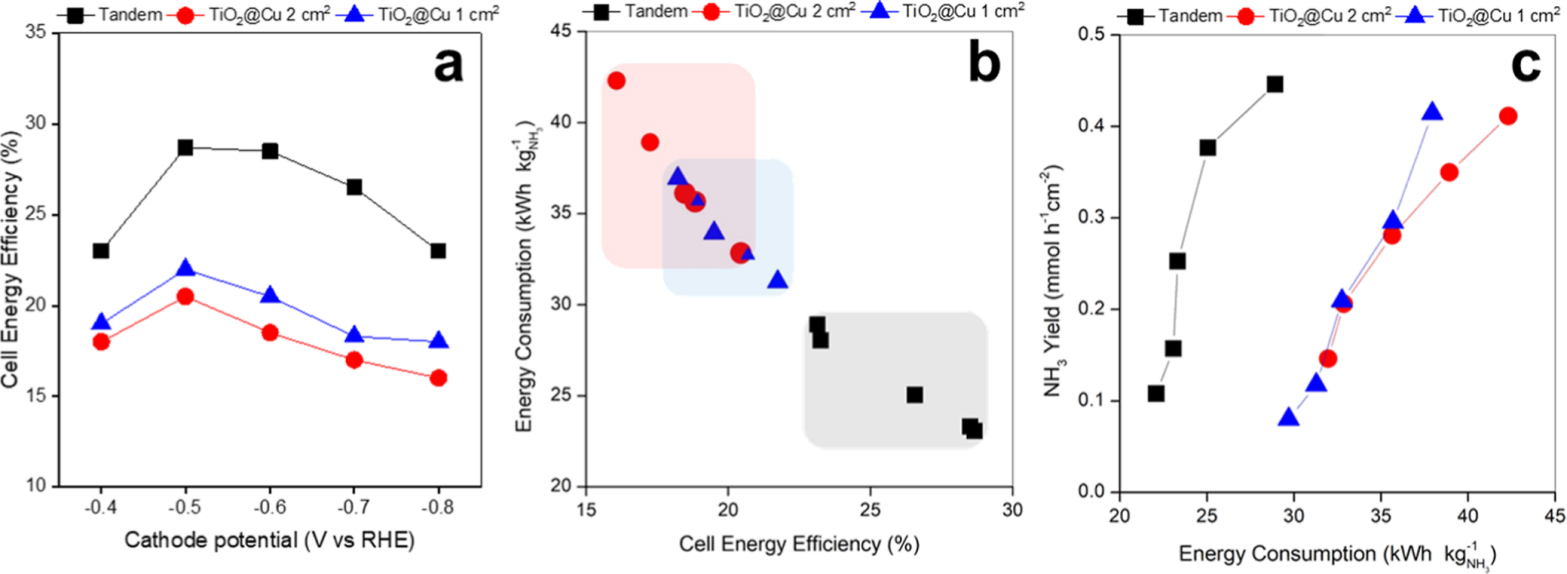
(a) EE_cell_ as a function of *E*_W_; (b) variation of EC with respect to EE_cell_, and (c) NH_3_ productivity as a function of EC per kg of NH_3_ produced.

Figure 9b shows the relationship between EC_NH_3__ and EE_cell_ for the three configurations. EC_NH_3__, expressed in kWh kg^–1^, quantifies the energy required to produce 1 kg of NH_3_. For the 1 cm^2^ and 2 cm^2^ TiO_2_@Cu electrodes, EC_NH_3__ ranged from 43 kWh kg^–1^ at the lowest EE_cell_ value (16%) to 30 kWh kg^–1^ at the highest (20%), with intermediate data points overlapping (red and blue regions). The tandem configuration achieved EC_NH_3__ values between 29 kWh kg^–1^ (at EE_cell_ = 23%) and 23 kWh kg^–1^ (at EE_cell_ = 28%), demonstrating its superior energy performance.

For context, the minimum theoretical energy requirement for NH_3_ production, given by its lower heating value, is 5.92 kWh kg^–1^, while the minimum required for the Haber-Bosch process ranges from 16.7 to 8.8 kWh kg^–1^, depending on the technology used.^58^ The tandem system, therefore, represents an energetically favorable approach for the electrochemical NO_3_^–^ to NH_3_ conversion. This improvement is attributed to the lower activation energy for the NO_3_^–^ → NO_2_^–^ step on the Cu surface, which provides kinetic benefits and ultimately enhances the overall NO_3_^–^RR to NH_3_ efficiency.

The decrease in EC_NH_3__ directly impacts the energy requirements for achieving higher productivities. As shown in Figure 9c, the tandem system consistently attains higher NH_3_ productivities at lower EC_NH_3__ values compared to 1 cm^2^ and 2 cm^2^ TiO_2_@Cu cathodes. For example, to reach a productivity of ∼0.1 mmol h^–1^ cm^–2^, the tandem configuration requires only 22.10 kWh kg^–1^, whereas the single- and double-sided TiO_2_@Cu electrodes demand 30–32 kWh kg^–1^. Increasing the working potential from −0.4 to −0.7 V vs RHE enhanced the tandem productivity by ∼ 3.5x (up to 0.38 mmol h^–1^ cm^–2^) while raising EC_NH_3__ by only 2.5 kWh kg^–1^, consistent with the EE_cell_ vs *E*_W_ trends. However, further increasing productivity to 0.44 mmol h^–1^ cm^–2^ (at −0.8 V vs RHE) required an EC_NH_3__ of 29 kWh kg^–1^, reflecting the drop in EE decrease at high overpotentials due to HER and other competing reactions. By contrast, the 1 cm^2^ and 2 cm^2^ TiO_2_@Cu configurations require 38–43 kWh kg^–1^ to 0.41 mmol h^–1^ cm^–2^, representing over a 40% increase in energy consumption with respect to the tandem approach.

### 3.5 Scaling-Up Parameters—Galvanostatic Conditions

Figure 10 summarizes the performance of the tandem configuration under galvanostatic operation at constant current densities from 35 mA to 110 mA cm^–2^. The FE_NH_3__ exhibits a clear volcano-shaped trend, peaking at 97% at 90 mA cm^–2^ and maintaining values above 80% across all tested conditions. The corresponding potentials at the highest FE (−0.7 and −0.8 V vs RHE for 70 and 90 mA cm^–2^, respectively) differ slightly from potentiostatic tests (Figure S5b), where the maximum FE was observed at −0.6 V vs RHE. The highest FE toward NO_2_^–^ occurred at the lowest current density.

**Figure 10:**
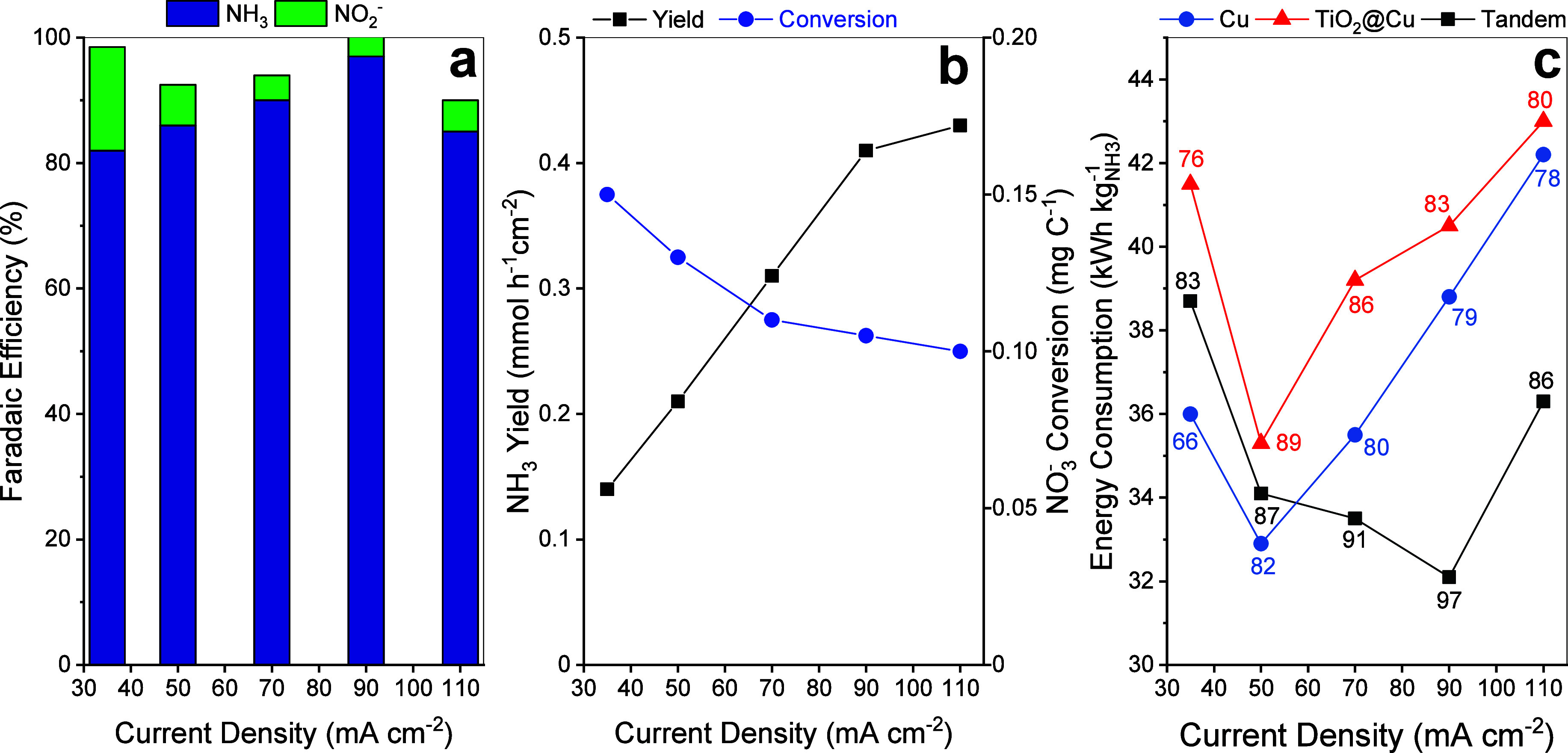
(a) FE distribution, (b) NH_3_ productivities and specific NO_3_^–^ conversion, and (c) energy consumption per kg of NH_3_ at different current densities with the tandem approach. The numbers next to the points in c correspond to the FE_NH_3__.

NH_3_ productivity increased linearly with current density, reaching 0.43 mmol h^–1^ cm^–2^ at 110 mA cm^–2^, representing the apparent performance limit under these conditions. This limit likely arises from a transient depletion of available NO_3_^–^, as up to 45% of the initial NO_3_^–^ was continuously converted (Figure S7a). Indeed, the specific NO_3_^–^ conversion per unit charge decreased with current density, from 0.15 to 0.10 mg C^1–^, consistent with the lower limits observed in our previous Cu/Ti-based NH_3_ generation system.^34^

Energy consumption for producing 1 kg of NH_3_ (EC_NH_3__, kWh kg^–1^) was also evaluated for three cathode configurations: tandem, two-side Cu, and two-side TiO_2_@Cu (Figure 10c). For the single-material electrodes, the minimum energy consumption occurred at 50 mA cm^–2^, coinciding with their maximum FE. In contrast, the tandem system achieved its lowest energy requirement at 90 mA cm^–2^, where it also delivered 97% FE to NH_3_. Notably, the pristine Cu electrode exhibited low EC_NH_3__ at the low current densities but suffered from poor NH_3_ selectivity, whereas TiO_2_@Cu consumed more energy but provided a higher selectivity than Cu.

Besides the demonstration of the improved EE achieved with the tandem configuration, additional cycling experiments were performed to evaluate the stability of both the electrode and the overall system. The tests were conducted under identical experimental conditions, with a constant current density of 90 mA cm^–2^. Individual cycles were analyzed, and the electrolyte was replaced between cycles. As shown in Figure S8, stable cell voltages between 2.1 and 1.9 V were observed, together with a consistent FE of 92–95% and NH_3_ production rates of approximately 0.4 mmol h^–1^ cm^–2^.

Figure 11 shows $\Delta C_{NO_{3}^{-}}$ as a function of $EC_{NO_{3}^{-}}$ , offering a complementary figure of merit that quantifies the energy required to convert 1 kg of NO_3_^–^. This parameter is particularly valuable for estimating and comparing the operation costs of energy-intensive water treatment systems.^59^ Here, it is applied to benchmark the tandem configuration against various catalysts reported for electrochemical denitrification. Under potentiostatic conditions, the tandem system achieved an $EC_{NO_{3}^{-}}$ as low as 2.89 kWh kg^–1^ cm^–2^ and 4.95 kWh kg^–1^ cm^–2^ under galvanostatic operation, representing the lowest reported values among recent studies. By contrast, other Cu-based catalysts, such as Cu/Ni^60^ or Cu/Zn,^61^ exhibited higher specific NO_3_^–^ conversions but at substantially greater energy costs. It should be noted that some literature values correspond to systems operating under different conditions (e.g., lower initial NO_3_^–^ concentrations), which contributes to the widespread of $EC_{NO_{3}^{-}}$ values shown in the figure.

**Figure 11:**
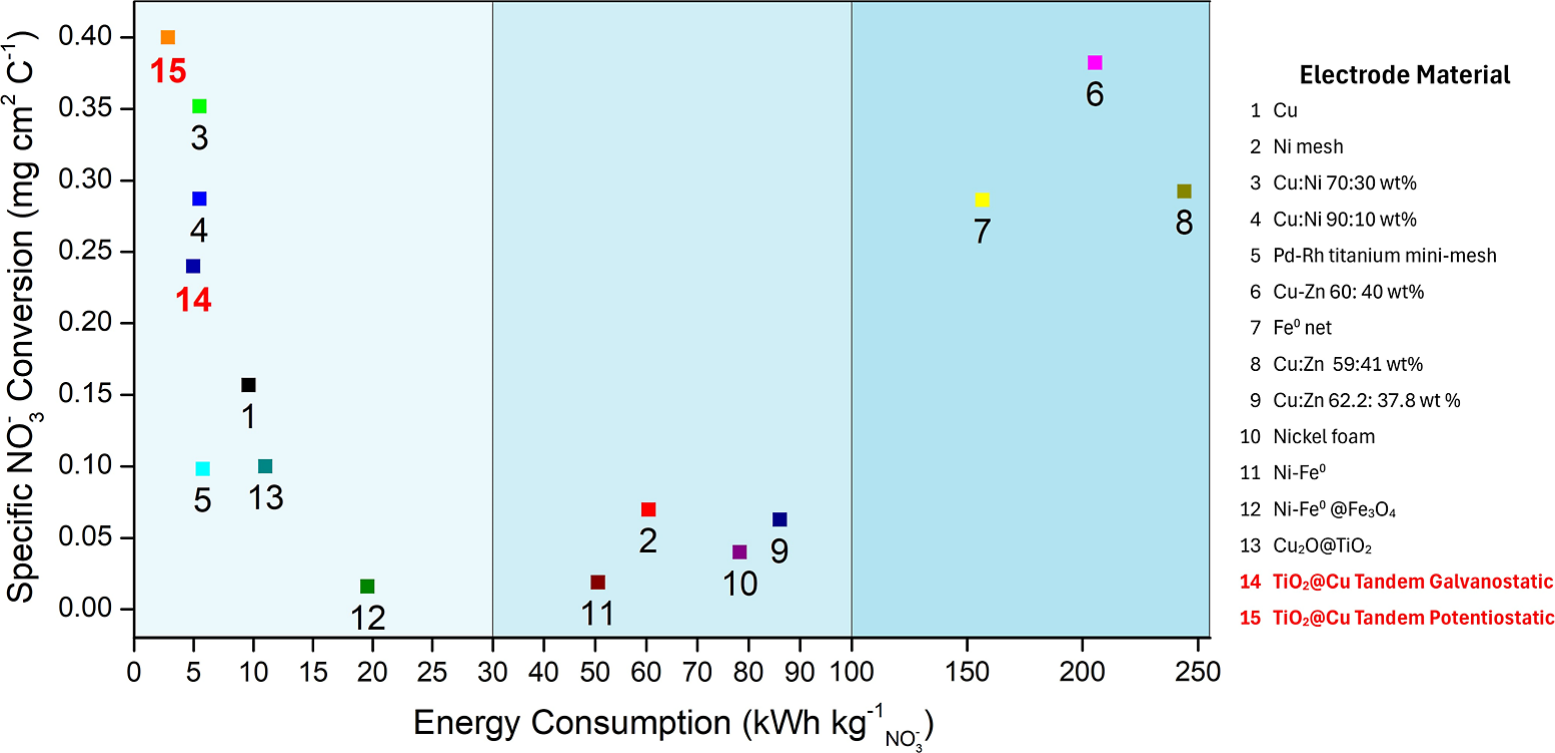
Specific NO_3_^–^ conversion by unit charge as a function of the energy consumption to convert 1 kg of NO_3_^–^ for different electrode materials used in NO_3_^–^RR, and a comparative with other reported values in literature.

Overall, the outstanding performance of the tandem configuration highlights its potential for energy-efficient denitrification, either for complete NO_3_^–^ removal from water or for NH_3_ as a production as the main product. These results provide preliminary scaling parameters that indicate significantly reduced energy requirements compared to conventional approaches. Future mechanistic studies aimed at elucidating the role of each individual surface and the influence of operating conditions could further guide the optimization of the electrode architecture.

## 4 Conclusion

In summary, we demonstrate a cascade electrolysis strategy for the conversion of nitrate to ammonia in alkaline media, employing a tandem system with two distinct active surfaces, Cu and TiO_2_@Cu, operating under identical polarization conditions. This configuration yields markedly enhanced energy efficiencies for ammonia production, with energy consumption reduced by more than 32% compared to single-surface TiO_2_@Cu electrodes, particularly at moderate applied potentials as low as −0.7 V. Mechanistic investigations reveal that Cu preferentially accelerates the rate-determining step nitrate-to-nitrite conversion, while TiO_2_@Cu excels in nitrite-to-ammonia hydrogenation, thereby reducing the overall overpotential for global NO_3_^–^RR. Energy consumption analysis further highlights the competitiveness of the tandem cascade approach, offering lower energy requirements than comparable systems.

Overall, this work establishes cascade cell configurations as an effective platform for tandem catalysis of reactive intermediates across spatially distinct active sites, significantly boosting the EE. Beyond advancing sustainable ammonia electrosynthesis, our findings exemplify how rational electrode and cell design can overcome intrinsic scaling relations in electrocatalysis and inspire innovative pathways for other multielectron reactions.

## Supplementary Material



## Notes

Additional experimental details on electrode preparation, cell configuration, analytical results, and efficiency calculations, additional results: EDX analysis, EPR spectroscopy, and XRD analysis, and additional results of galvanostatic and cycling tests (PDF)

## #

M.E. Chávez: The Institute of Photonic Sciences (ICFO), Mediterranean Technology Park, 08860 Castelldefels (Barcelona), Spain. Email: marcelo.chavez@icfo.eu

## ¶

S. Murcia-López: Catalan Institute of Nanoscience and Nanotechnology (ICN2), Edifici ICN2, UAB Campus, 08193 Bellaterra (Barcelona), Spain. Email: sebastian.murcia@icn2.cat.
