# Supplementary material for: Single-Electrode Tandem Electrocatalysis with Dual TiO2@Cu and Cu Surfaces Enables Energy-Efficient Ammonia Production from Nitrate
Source: ACS Sustain Chem Eng. 2026 Apr 23;14(17):8148–59. doi: 10.1021/acssuschemeng.5c14004 (PMC13147331; doi:10.1021/acssuschemeng.5c14004)
Supplement: Supplementary file 1 [file sc5c14004_si_001.pdf]

Supporting Information

Single-Electrode Tandem Electrocatalysis with Dual TiO_2@Cu and Cu Surfaces Enables Energy-Efficient Ammonia Production from Nitrate

Marcelo E. Chávez[a,b*#], Inna Y. Khairani[c], Martí Biset-Peiró[a], Sara Martí-Sánchez[d], Katherine Villa[e,f], Jordi Arbiol[d,f], Joan R. Morante[a,b], Bilal Gökce[c], Sebastián Murcia-López[a*‡]

[a] Catalonia Institute for Energy Research (IREC), Sant Adrià de Besòs 08930, Spain

[b] University of Barcelona (UB), Barcelona 08028, Spain

[c] Chair of Materials Science and Additive Manufacturing, School of Mechanical Engineering and Safety Engineering, University of Wuppertal, Wuppertal 42119, Germany

[d] Catalan Institute of Nanoscience and Nanotechnology (ICN2), CSIC and BIST, Campus UAB Bellaterra, 08193 Barcelona, Catalonia, Spain

[e] Institute of Chemical Research of Catalonia (ICIQ-CERCA), The Barcelona Institute of Science and Technology (BIST), Av. Països Catalans, 16, Tarragona E-43007, Spain

[f] ICREA, Pg. Lluís Companys 23, 08010 Barcelona, Catalonia, Spain

Number of pages: 12
Number of figures: 8

TABLE OF CONTENT

Figure S1. TiO_2@Cu electrode preparation. 3

Figure S2. Full electrochemical set-up: a) 1 cm^2 configuration; b) 2 cm^2 configuration. 4

Figure S3. a) NO_2^-/NO_3^- and b) NH_3/NH_4^+ calibration curves. 5

Figure S4. EDX mapping of TiO_2@Cu electrode with the corresponding elemental maps. 6

Figure S5. XRD results of the TiO_2@Cu electrode before and after the electrolysis tests. 7

Figure S6. EPR spectrum of TiO_2 nanoparticles. 7

Half-Cell and Global Cell Energy Efficiency determination. 8

Figure S7. a) NO_3^- conversion; b) E_w and E_{cell} in the tests under galvanostatic conditions. 10

Figure S8. Results of the cycling tests with the tandem configuration under galvanostatic conditions at 90 mA cm^{-2}: a) variation of the cell voltage; b) Faradaic efficiencies and NH_3 Yields. 11

Figure S1. TiO_2@Cu electrode preparation.

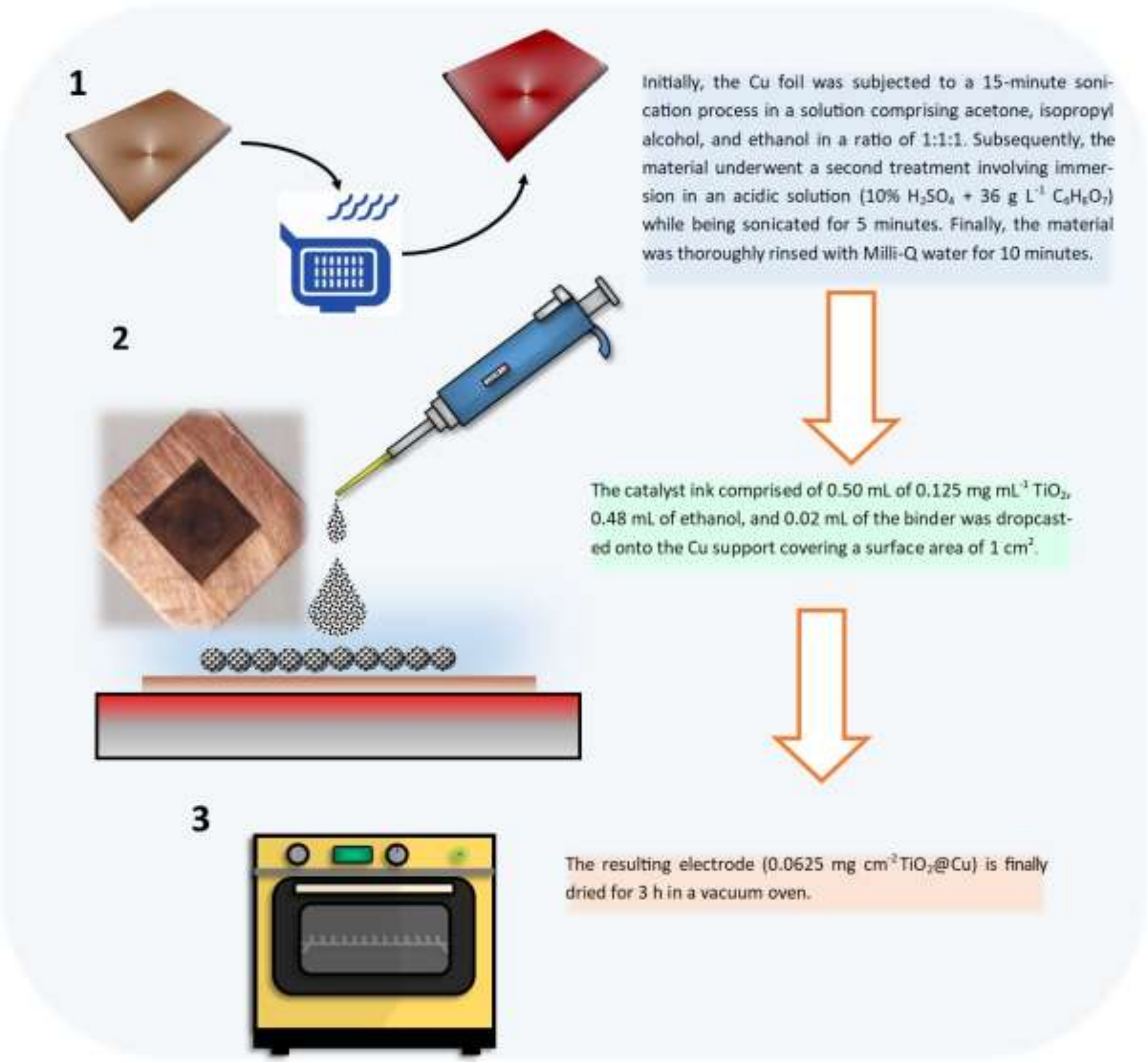

Figure S2. Full electrochemical set-up: a) 1 cm^2 configuration; b) 2 cm^2 configuration.

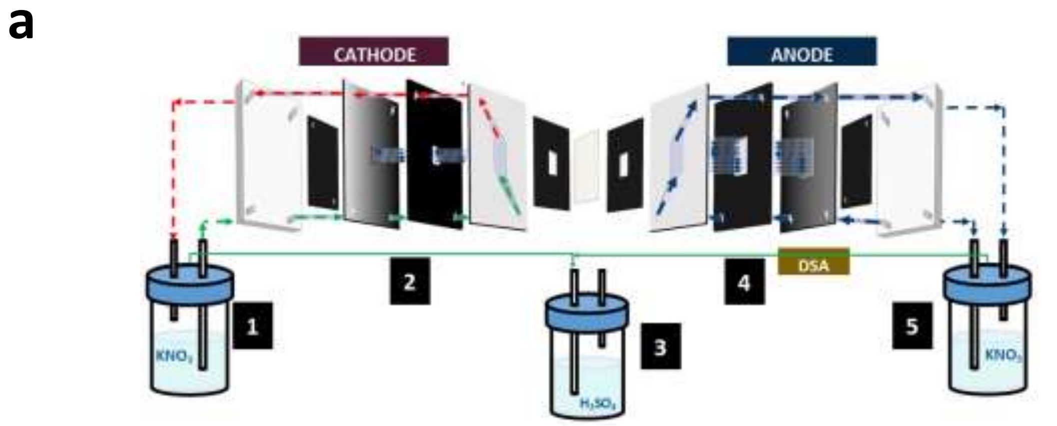

1) Catholyte container
2) Cathode TiO_2@Cu or Cu
3) Ammonia trap
4) DSA Anode
5) Anolyte container

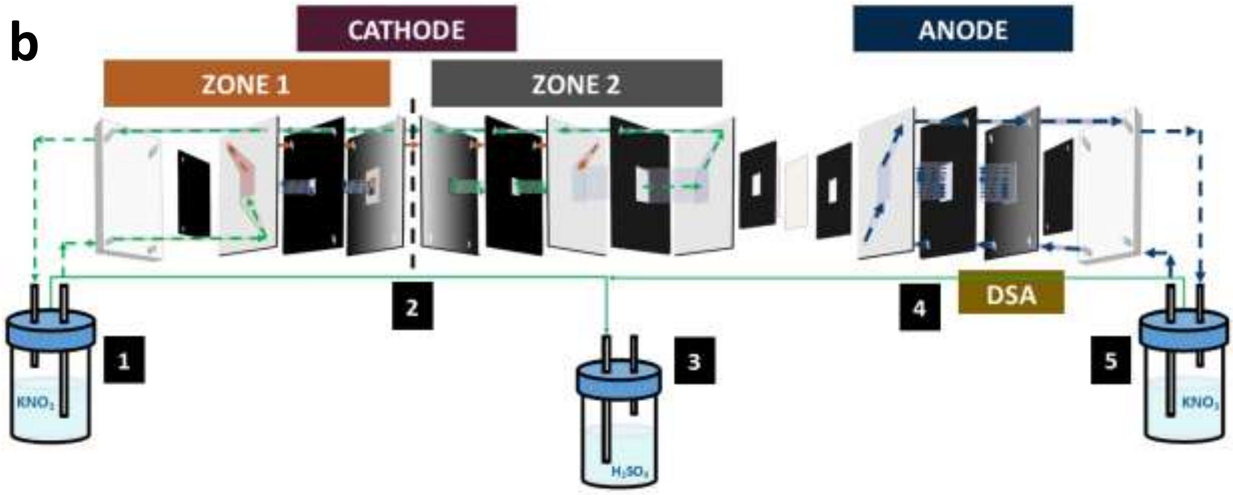

1) Catholyte container
2) Cathode Cu (Zone 1) / TiO_2@Cu (Zone 2)
3) Ammonia trap
4) Dimensionally stable anode
5) Anolyte container

Figure S3. a) NO_2^-/NO_3^- and b) NH_3/NH_4^+ calibration curves.

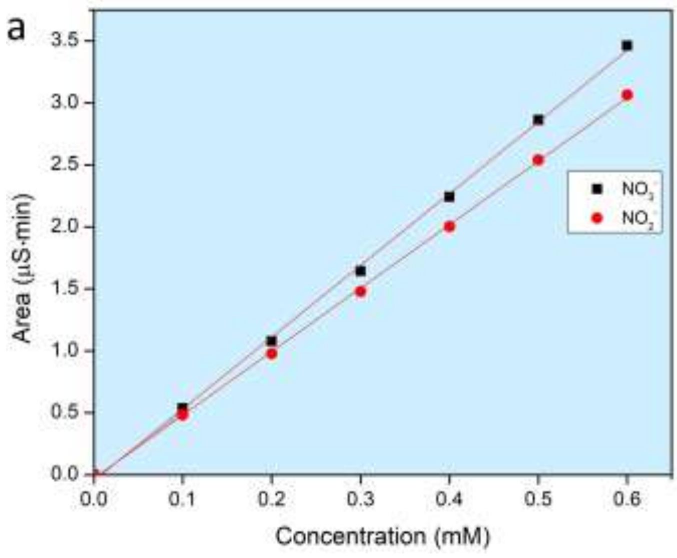

Calibration curves for NO_3^- and NO_2^- anions were established by correlating peak areas (µS·min) with the concentration of standard solutions (ranging from 0.0 to 0.6 mM for each ion). The mobile phase (eluent) used for anion quantification comprised 4.5 mM sodium carbonate (Na_2CO_3) and 1.4 mM sodium hydrogen carbonate ($NaHCO_3$), with a flow rate of 1.5 mL·min^{-1}. To measure the concentration of samples, a 55 µL aliquot of electrolyte was diluted in 10 mL of Millipore water and subsequently analyzed using IC. The actual ion concentration in the electrolyte was determined by multiplying the measured concentration by the dilution factor (10/0.055).

$$A = 5052.4 \cdot C_{NO_2^-}$$
$$A = 5680.2 \cdot C_{NO_3^-}$$

A [µS·min]
C [mM]

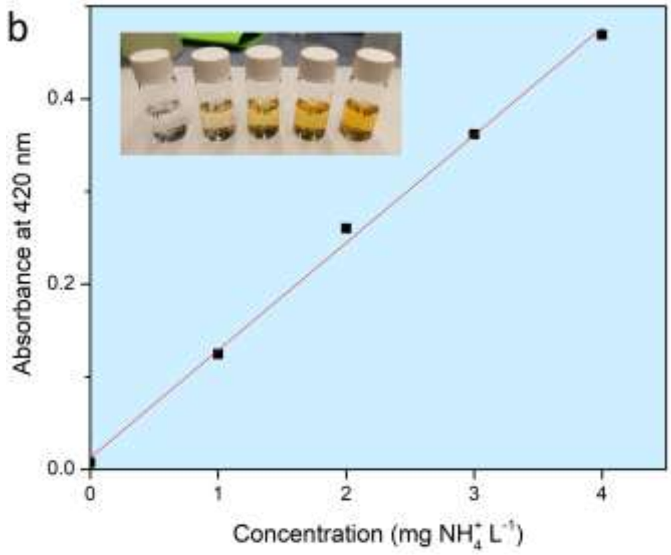

For NH_3/NH_4^+ quantification, a calibration curve was constructed using five standard concentrations of ammonium chloride (NH_4Cl): 0, 0.06, 0.11, 0.17, and 0.22 mM (0 to 4 mg L^{-1} of NH_4^+ respectively). Each sample's NH_3/NH_4^+ concentration was determined by diluting an aliquot 100 to 200 times (0.050 to 0.100 mL up to 10 mL) with Millipore water to match the calibration curve's concentration range. To each diluted sample (10 mL), two drops of Polyvinyl alcohol dispersing agent and two drops of a mineral stabilizer solution were added.
The formation of the complex compound Hg_2ONH_2I was observed, and its absorbance was measured at 420 nm within 10 to 30 minutes following the addition of the Nessler reagent.

$$Abs = 0.1203 \cdot C_{NH_3}$$

Abs [Dimensionless]
C [mg L^{-1}]

Figure S4. EDX mapping of TiO_2@Cu electrode with the corresponding elemental maps.

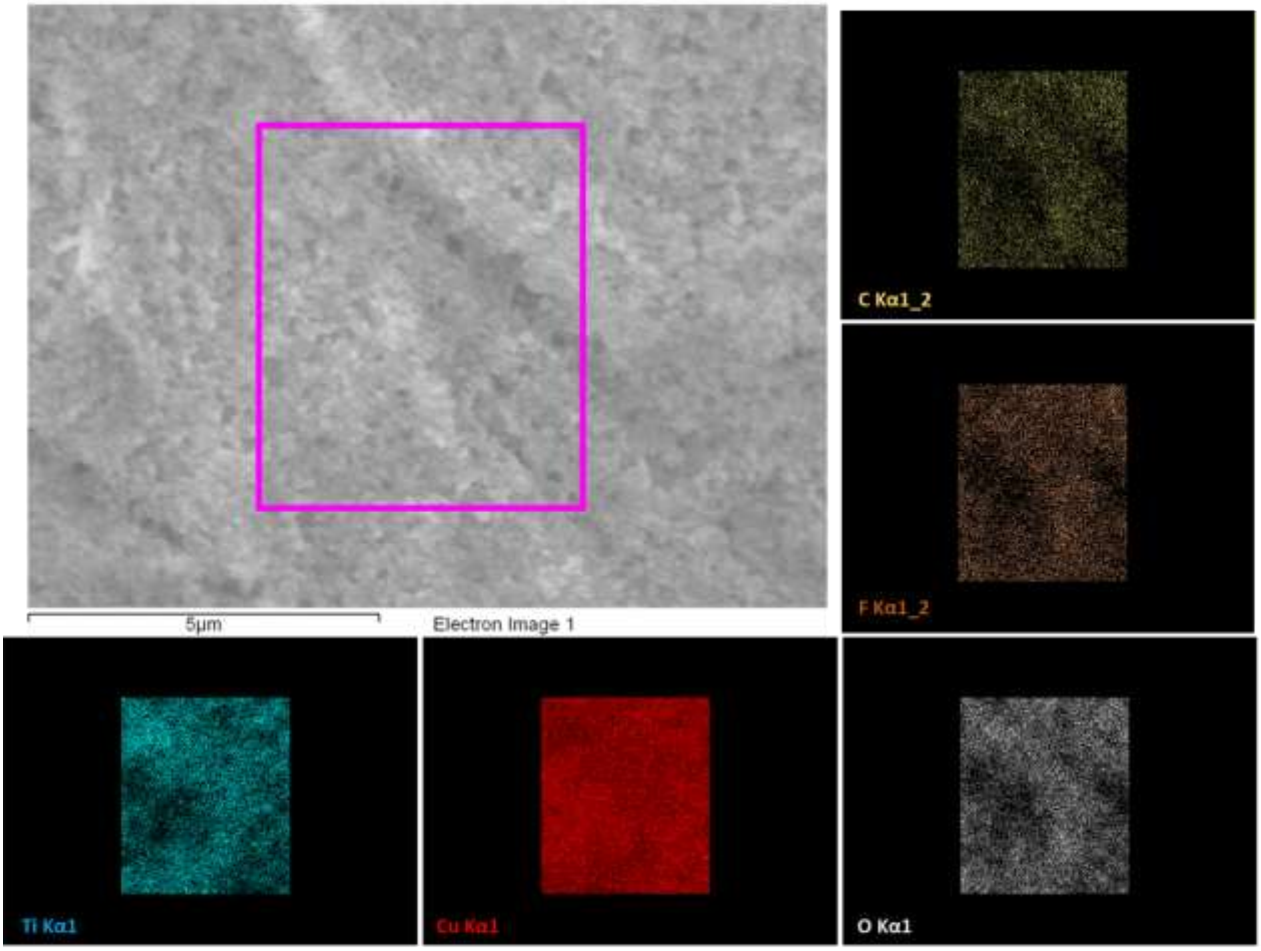

Figure S5. XRD results of the TiO_2@Cu electrode before and after the electrolysis tests.

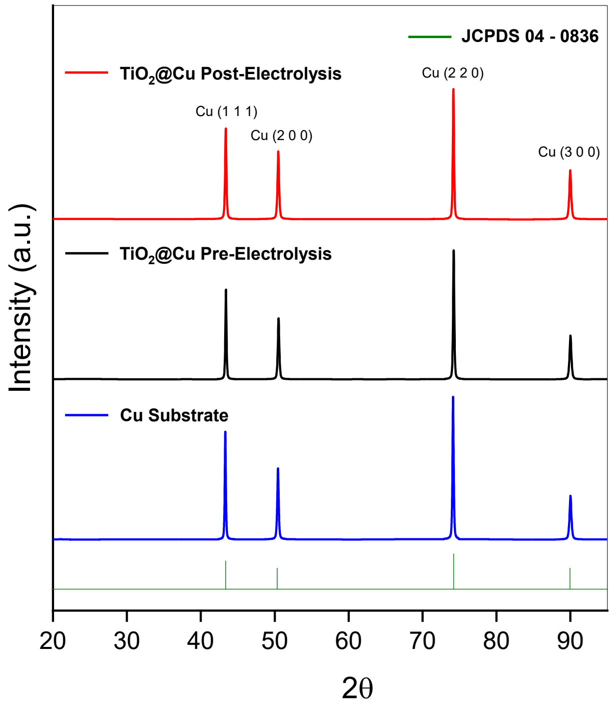

Figure S6. EPR spectrum of TiO_2 nanoparticles.

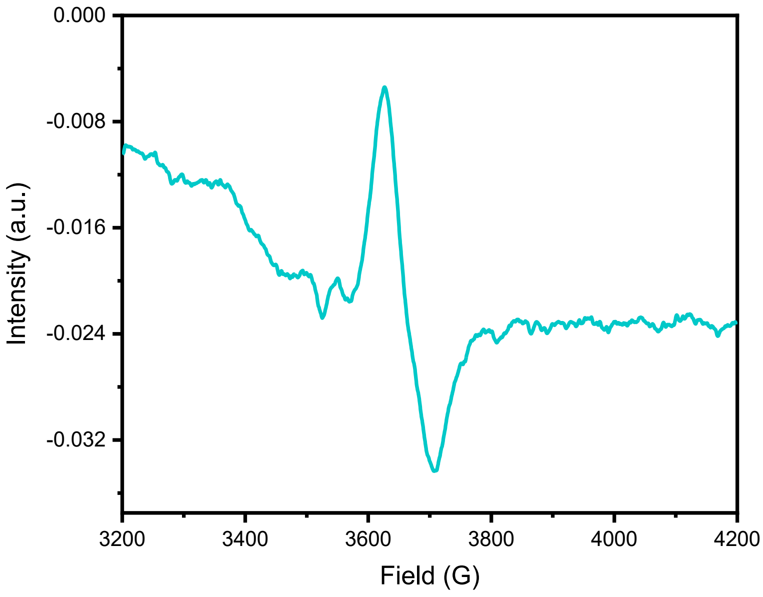

Half-Cell and Global Cell Energy Efficiency determination.

1- The Cell Energy efficiency (EE_{CELL}) of the system defines the percentage with which input energy is used for converting NO_3^- and intermediates to NH_3 within the entire system. The EE_{CELL} would involve the energy input, chemical energy output, and losses due to inefficiencies in the electrochemical processes of flow cell operation. For this, the Energy Consumption (EC) corresponds to the theoretical necessary energy for NH_3 formation. Considering the reactions:

$NO_2^- + 5H_2O + 6e^- \rightarrow NH_3 + 7OH^-$	E°= -0.174V vs $SHE_{pH=14}$ [1]	CATHODE
$NO_3^- + 6H_2O + 8e^- \rightarrow NH_3 + 9OH^-$	E°= -0.132V vs $SHE_{pH=14}$ [1]	
$4OH^- \rightarrow 2H_2O + O_2 + 4e$	E°= 0.4V vs $SHE_{pH=14}$ [2]	ANODE

$$\boldsymbol{E^0_{cell} = E^0_{Red} - E^0_{Ox}}$$

$$\boldsymbol{\Delta G^0_{NH_3} = -nFE^0_{CELL}}$$

$$\boldsymbol{EC_{NH_3} = \left[\Delta G^0_{NH_3} + R \cdot T \cdot \mathrm{Ln}\left(\frac{C_{NH_{3t}} \cdot C_{OH^-{}_t}^{9}}{C_{NO_x^-{}_t}}\right)\right] \cdot C_{NH_{3t}} \cdot V}$$

$$\boldsymbol{EE_{cell}}(\%) = \frac{\boldsymbol{|EC_{NH_3}|}}{\boldsymbol{|E_{CELL} \cdot Q|}} \boldsymbol{\cdot 100}$$

Where:

$\Delta G^0_{NH_3}$ = Standard Gibbs Free Reaction Energy ($J \cdot mol^{-1}$)

E^0_{CELL} = Standard Cell Potential (V)

R = Universal gas constant ($J \cdot mol^{-1} \cdot K^{-1}$)

T = Temperature (K)

F = Faraday Constant ($C \cdot mol^{-1}$)

n = Number of transferred electrons

Q = Transferred charge

2- The Half-Cell Energy Efficiency ($EE_{half\text{-}cell}$) measures how efficiently the electrical energy input is converted into the chemical energy stored in NH_3 only considering the half-cell reaction in the cathode.

$$\boldsymbol{EE_{half-cell}}(\%) = \frac{\boldsymbol{EC_{NH_3}}}{\boldsymbol{(0.4 - E_w) \cdot Q}} \boldsymbol{\cdot 100}$$

Where:

E_w = Applied Potential (V)

The overpotential of anodic OER is assumed to be zero.

For the Figure 6b. Electrode TiO_2@Cu during NO_2^-RR.

$$NO_2^- + 5H_2O + 6e^- \rightarrow NH_3 + 7OH^- \qquad E^\circ = -0.174V \text{ vs } SHE_{pH=14}$$ [1]

$\Delta G^\circ_{NH_3}$= 332.3 kJ mol^{-1} [3] E_W= -0.6V vs RHE (-1.426V vs SHE) Q= 304 C

$C_{NO_2^-{}_0}$= 0.1101 M $C_{NO_2^-{}_t}$ =0.0871M $C_{NH_3\,t}$ = 0.0210 M V= 0.025 L C_{OH^-} = 1M

$$EC_{NH_3}(J) = \left[332294\frac{J}{mol} + 8.314\frac{J}{mol\cdot K} \cdot 298K \cdot \text{Ln}\left(\frac{0.0210\cdot 1^9}{0.0871}\right)\right] \cdot 0.0210\frac{mol}{L} \cdot 0.025\,L = 172 \text{ J}$$

$$EE_{half-cell}(\%) = \frac{|172\,J|}{|0.4-(-1.423)|\cdot 304} \cdot 100 = 31\%$$

For the Figure 7a. Electrode Cu/TiO_2@Cu during NO_3^-RR in tandem configuration.

$$NO_3^- + 6H_2O + 8e^- \rightarrow NH_3 + 9OH^- \qquad E^\circ = -0.132 \text{ V vs } SHE_{pH=14}$$ [1]

$$4OH^- \rightarrow 2H_2O + O_2 + 4e \qquad E^\circ = 0.4 \text{ V vs } SHE_{pH=14}$$ [2]

$\Delta G^\circ_{NH_3}$= 410.64 kJ mol^{-1} [3] E_W= -0.6V vs RHE (-1.423V vs SHE) E_{cell}= 2.23V Q= 259 C

$C_{NO_3^-{}_0}$= 0.1080 M $C_{NO_3^-{}_t}$ =0.0821 $C_{NH_3\,t}$ = 0.0162 M V= 0.025 L C_{OH^-} = 1M

$$EC_{NH_3}(J) = \left[410640\frac{J}{mol} + 8.314\frac{J}{mol\cdot K} \cdot 298K \cdot \text{Ln}\left(\frac{0.0162\cdot 1^9}{0.0821}\right)\right] \cdot 0.0162\frac{mol}{L} \cdot 0.025\,L = 165 \text{ J}$$

$$EE_{cell}(\%) = \frac{|165|}{|259\cdot 2.23|} \cdot 100 = 29\%$$

Figure S7. a) NO_3^- conversion; b) E_w and E_{cell} in the tests under galvanostatic conditions.

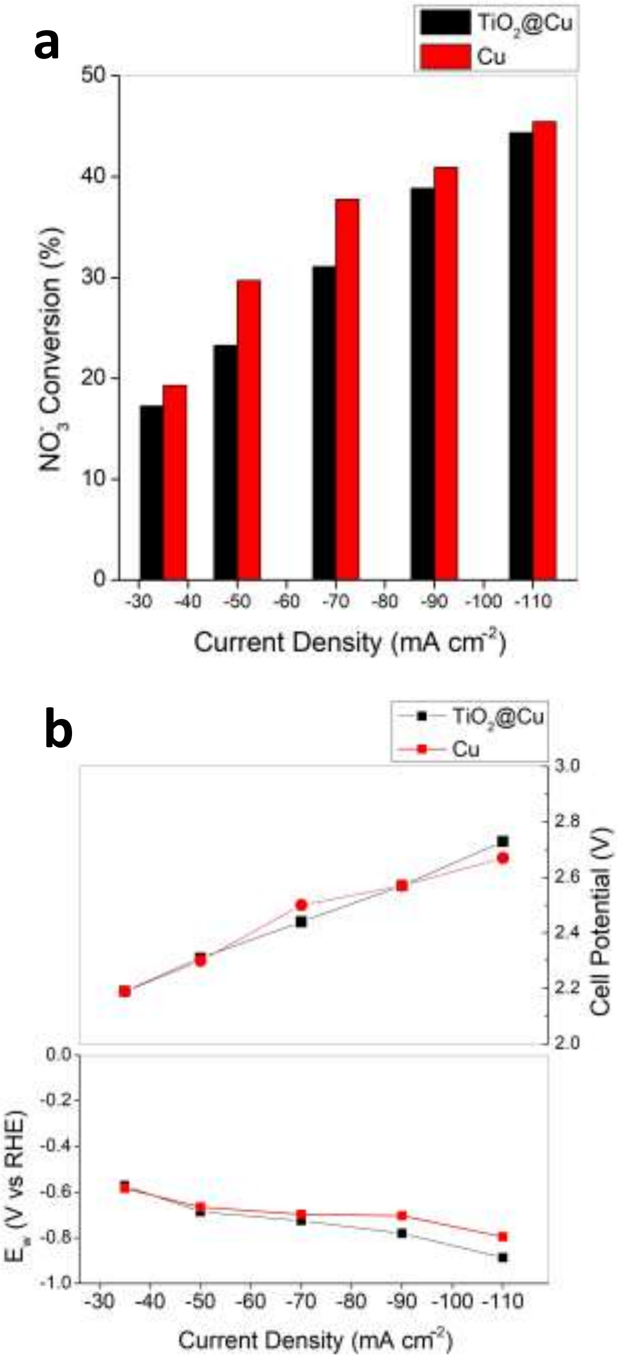

Figure S8. Results of the cycling tests with the tandem configuration under galvanostatic conditions at 90 mA cm^{-2}: a) variation of the cell voltage; b) Faradaic efficiencies and NH_3 Yields.

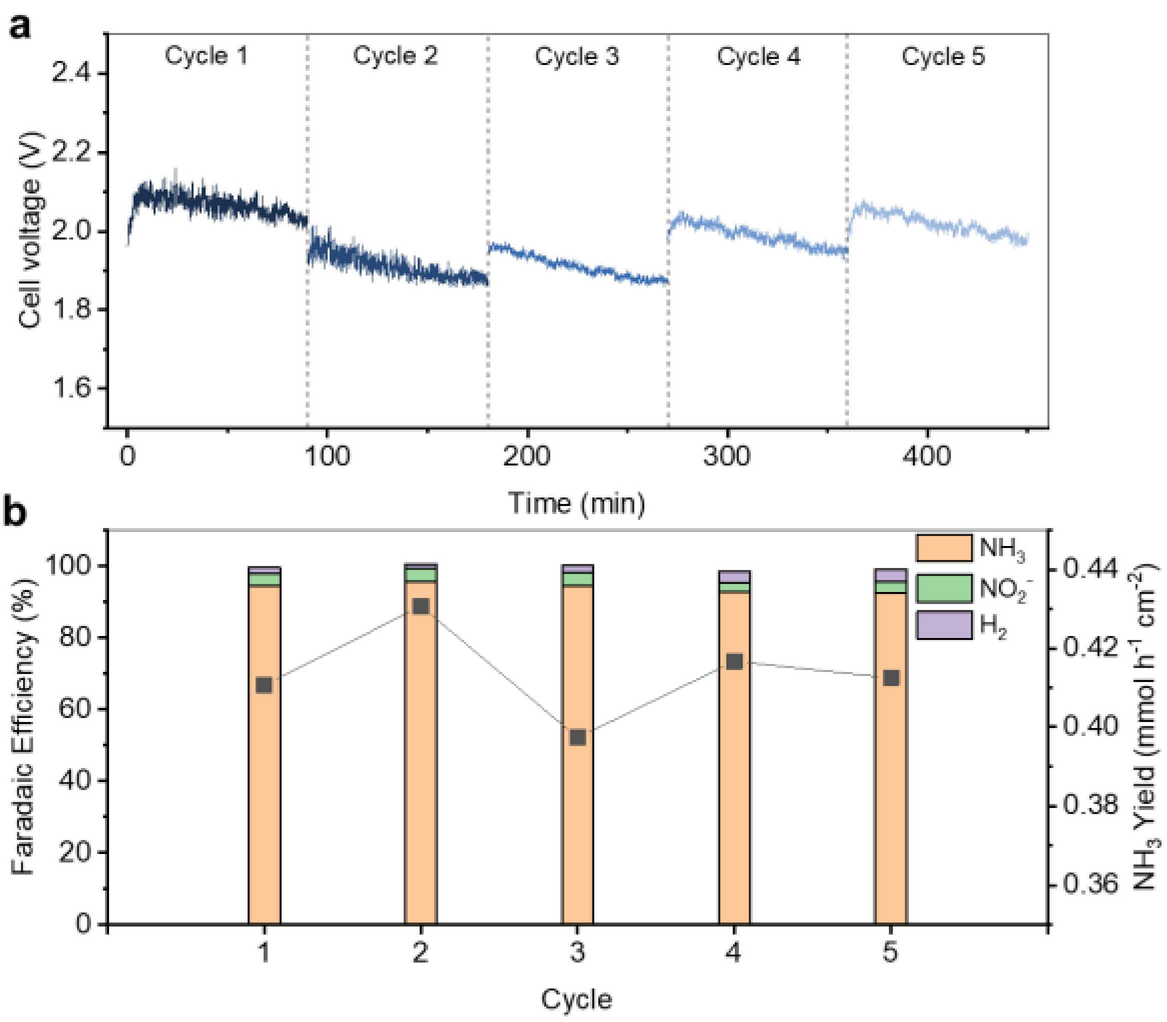

REFERENCES

1. Choueiri, R. M.; Tatarchuk, S. W.; Klinkova, A.; Chen, L.D. Mechanism of ammonia oxidation to dinitrogen, nitrite, and nitrate on β-$Ni(OH)_2$ from first-principles simulations. *Electrochem. Sci. Adv.* **2022**, 2, 1.

2. Govind Rajan, A.; Carter, E.A. Microkinetic model for pH- And potential-dependent oxygen evolution during water splitting on Fe-doped β-NiOOH. *Energy Environ. Sci.* **2020**, 13, 4962.

3. Dolfing, J.; Hubert, C.R.J. Using thermodynamics to predict the outcomes of nitrate-based oil reservoir souring control interventions. *Front. Microbiol.* **2017**, 8, 1.
